# Metabolic Cycles in Yeast Share Features Conserved among Circadian Rhythms

**DOI:** 10.1016/j.cub.2015.02.035

**Published:** 2015-04-20

**Authors:** Helen C. Causton, Kevin A. Feeney, Christine A. Ziegler, John S. O’Neill

**Affiliations:** 1 Department of Biological Sciences, Columbia University, 617 Fairchild Building, 1212 Amsterdam Avenue, Mail Code 2442, New York, NY 10027, USA; 2 MRC Laboratory of Molecular Biology, Francis Crick Avenue, Cambridge CB2 0QH, UK

## Abstract

Cell-autonomous circadian rhythms allow organisms to temporally orchestrate their internal state to anticipate and/or resonate with the external environment [1, 2]. Although ∼24-hr periodicity is observed across aerobic eukaryotes, the central mechanism has been hard to dissect because few simple models exist, and known clock proteins are not conserved across phylogenetic kingdoms [1, 3, 4]. In contrast, contributions to circadian rhythmicity made by a handful of post-translational mechanisms, such as phosphorylation of clock proteins by casein kinase 1 (CK1) and glycogen synthase kinase 3 (GSK3), appear conserved among phyla [3, 5]. These kinases have many other essential cellular functions and are better conserved in their contribution to timekeeping than any of the clock proteins they phosphorylate [6]. Rhythmic oscillations in cellular redox state are another universal feature of circadian timekeeping, e.g., over-oxidation cycles of abundant peroxiredoxin proteins [7–9]. Here, we use comparative chronobiology to distinguish fundamental clock mechanisms from species and/or tissue-specific adaptations and thereby identify features shared between circadian rhythms in mammalian cells and non-circadian temperature-compensated respiratory oscillations in budding yeast [10]. We find that both types of oscillations are coupled with the cell division cycle, exhibit period determination by CK1 and GSK3, and have peroxiredoxin over-oxidation cycles. We also explore how peroxiredoxins contribute to YROs. Our data point to common mechanisms underlying both YROs and circadian rhythms and suggest two interpretations: either certain biochemical systems are simply permissive for cellular oscillations (with frequencies from hours to days) or this commonality arose via divergence from an ancestral cellular clock.

## Highlights

• Yeast respiratory oscillations (YROs) share features with circadian rhythms
• Changes that alter the period of circadian rhythms have the same effect on YROs
• Oxidation cycles of peroxiredoxins are a characteristic of both oscillations
• Mechanistic similarities between these cycles may reflect a common origin

## Teaser

The clock gene feedback loops thought to drive circadian rhythms are not conserved across eukaryotes, but, perplexingly, several enzymes that determine clock speed are. Causton et al. now show that respiratory cycles in yeast share some key features with the clock in mammalian cells, raising questions about the origins of biological timekeeping.

## Results and Discussion

### A Conserved Cell-Cycle Regulator, Swe1, Also Regulates the YRO

**Figure 1:**
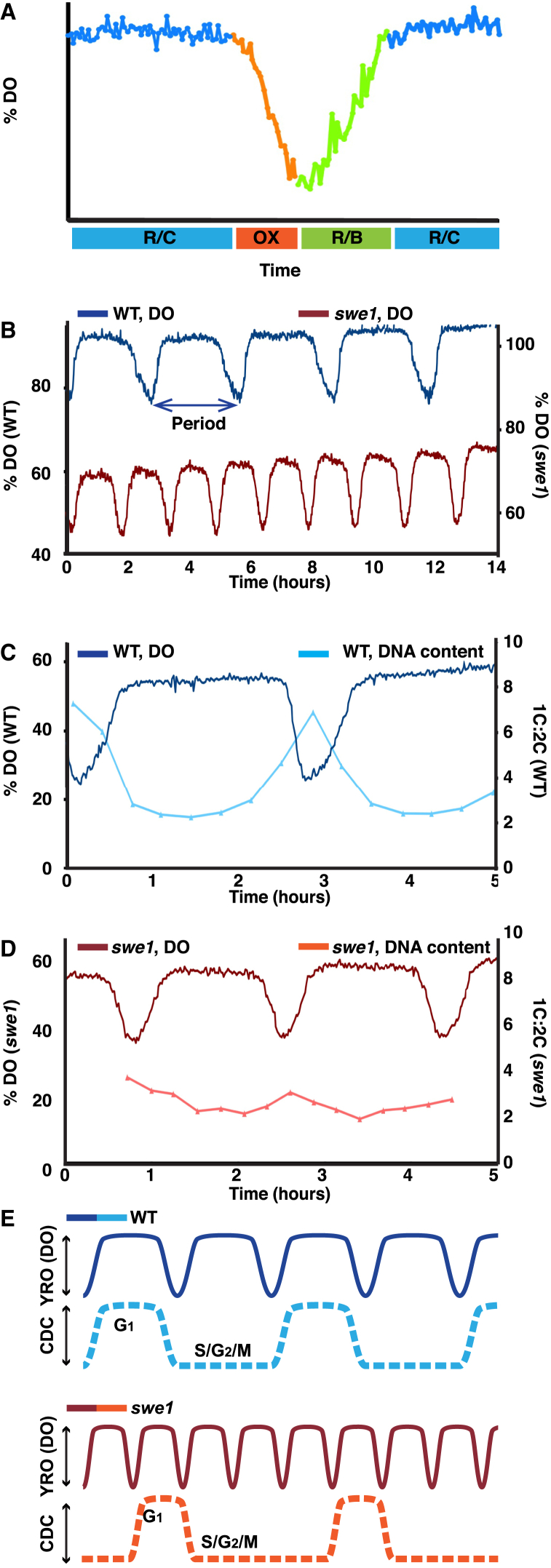
The CDC and YROs Are Affected by Deletion of *SWE1* (A) Phases of the oscillation referred to throughout the text: reductive/charging (R/C), respiratory or oxidative (OX), and reductive building (RB). (B) Dissolved oxygen (DO) trace showing that wild-type strains cycle with YRO (period 2.77 hr, SD 0.26, n = 17), whereas *swe1* strains cycle substantially faster (t test, p < 0.001; period 1.62 hr, SD 0.11, n = 12). See also Figure S1. (C and D) Dissolved oxygen traces are highly synchronized with DNA content in wild-type strains and *swe1* strains. (E) Model inferred from population-based data illustrating the relationship between the YRO and the CDC for wild-type and *swe1* strains. The dotted line represents the CDC of a single cell within the population. The population is not synchronized with respect to cell division, but the YRO gates when a cell can enter S phase.

In order to understand why certain ubiquitous post-translational mechanisms have a highly conserved circadian clock function, we investigated their contribution to the shorter period (∼1–5 hr, ultradian) yeast respiratory oscillations (YROs) in *Saccharomyces cerevisae*, which lack robust circadian rhythms and canonical clock proteins [11]. The YRO is a cell-autonomous, temperature-compensated rhythm in oxygen consumption that synchronizes spontaneously when cells are grown at high density in aerobic, nutrient-limited, continuous culture [10, 12–14] (Figure 1A). Beyond the difference in oscillation frequency (∼1 day^−1^ versus ∼8 day^−1^), circadian rhythms and YROs are highly similar (summarized in Table S1). In animal cells, the circadian clock regulates the timing of cell division [15], and in rapidly proliferating cells, the cell division cycle (CDC) and circadian cycle can become tightly coupled [16]. This is achieved, in part, through daily rhythms in the expression of a conserved cell-cycle regulator, the Ser/Thr kinase Wee1 [17, 18]. The homolog of Wee1 in budding yeast, Swe1, functions at the G2/M checkpoint, with additional roles during G_1_ [19]. Like the circadian cycle, the YRO does not require cell division [20] but does gate DNA replication so that S-phase entry does not typically occur during the oxidative portion of the YRO (OX) [21]. We asked whether coupling between the YRO and the CDC in yeast might occur via Swe1 in the same way that Wee1 connects circadian rhythms and the CDC in mammalian cells.

*SWE1* was deleted and synchronized respiratory oscillations were initiated in a bioreactor. The *swe1* strain underwent YROs with significantly shorter period than wild-type (Figures 1B–1D) but grew more slowly (44% ± 10% of wild-type; Figure S1D), confirming that the relationship between YRO and CDC is not fixed [20] and is regulated by Swe1. The proportion of G_1/0_ cells relative to cells with replicated DNA is represented by the 1C:2C ratio and peaks at the end of OX [12, 21]. The amplitude of 1C:2C across the oscillation was significantly attenuated in the mutant, with approximately half as many cells leaving G_1_ each oscillation compared with wild-type (Figures 1C, 1D, S1A, and S1B) and a higher percentage of cells in S/G_2_/M overall (Figure S1C). The YRO is specific to G_1/0_ [20], so it is likely that the faster respiratory oscillation of the *swe1* strain results from the relatively shorter duration of G_1/0_ in the mutant. Our data thus support a model in which the YRO and the CDC remain coupled in the *swe1* mutant, but the cell cycle is longer and fewer cells are undergoing respiratory oscillations at any point in time (Figure 1E). As mammalian cancer cell lines exhibit a wider range of circadian periods when assessed in vitro than do primary cells, we wonder whether altered circadian timekeeping might constitute a more general hallmark of cells that have lost tumor suppressor genes such as *WEE1*.

### CK1 and GSK3 Determine Period Length in Both the YRO and Mammalian Cellular Clock

**Figure 2:**
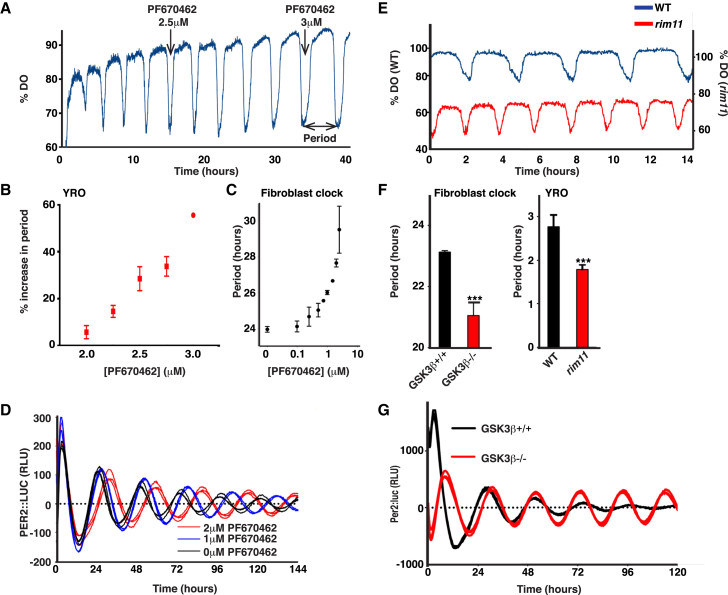
Perturbations that Affect the Period of Circadian Rhythms Have a Similar Effect on YROs (A) Representative DO traces showing that pharmacological inhibition of casein kinase I in yeast increases the period of oscillation. (B) Grouped data showing the dose dependence of CK1 inhibition on the YRO (mean ± SEM, one-way ANOVA, p = 0.0164). Although the target of PF670462 has not been characterized in yeast, addition of LH846, another CK1 inhibitor, had a similar effect (Figure S2). These results suggest that the period increase is due to direct inhibition of yeast CK1. (C) Grouped data showing dose-dependent period lengthening on circadian period in immortalized mouse fibroblasts (mean ± SD, n = 4, p < 0.0001 for concentration effect by two-way ANOVA). (D) PF670462 has a dose-dependent effect on circadian period in mouse fibroblasts as reported previously. Representative detrended bioluminescence traces are shown. (E) Representative data DO traces showing that a yeast strain deleted for *RIM11*, homolog of GSK3, has a shorter period of oscillation. (F) Bar graph showing the effect (mean ± SEM) of homozygous deletion of GSK3β on the period of the circadian cellular oscillation in fibroblasts (n = 4) and deletion of *RIM11* on the YRO (n = 17); p < 0.001 by unpaired t test in both cases. (G) Homozygous deletion of GSK3β shortens circadian period in mouse embryonic fibroblasts. Representative detrended traces are shown. Asterisks represent different p value thresholds (throughout): ^∗^p < 0.05; ^∗∗^p < 0.01; ^∗∗∗^p < 0.001.

CK1 and GSK3 are two families of ubiquitous eukaryotic Serine/Threonine kinases that regulate a broad range of cellular processes, including metabolism, cell migration, and *wnt* signaling (Table S2). These enzymes act to regulate target protein stability, subcellular localization, and complex formation and can act synergistically with each other, e.g., in the regulation of β-catenin. Both kinases also play a conserved role in setting the speed of the circadian clock, although their targets are not conserved (Table S2) [22, 23]. We postulated that these enzymes similarly contribute to the speed of the YRO. We tested this by pharmacological inhibition of yeast CK1δ/ε homologs using the selective inhibitors PF670462 and LH846 [23, 24] and by knocking out yeast GSK3β homolog, *RIM11*. In parallel, we performed experiments with mouse fibroblasts expressing transcriptional or translational clock gene::luciferase (*per2:lu*c or PER2::LUC) reporters (Figures 2 and S2). In both cases, our hypothesis was confirmed: CK1 inhibition dose dependently increased the period of the YRO and the mammalian clock, whereas GSK3β knockout significantly shortened the period of both. These results show that perturbation of the yeast CK1 and GSK homologs has similar effects to those observed for circadian rhythms in diverse species. Although we do not yet know which kinase substrates are relevant, our data are consistent with a model in which they play similar roles.

### Oscillations in the Redox State of Peroxiredoxin Tsa1 Accompanies the YRO

**Figure 3:**
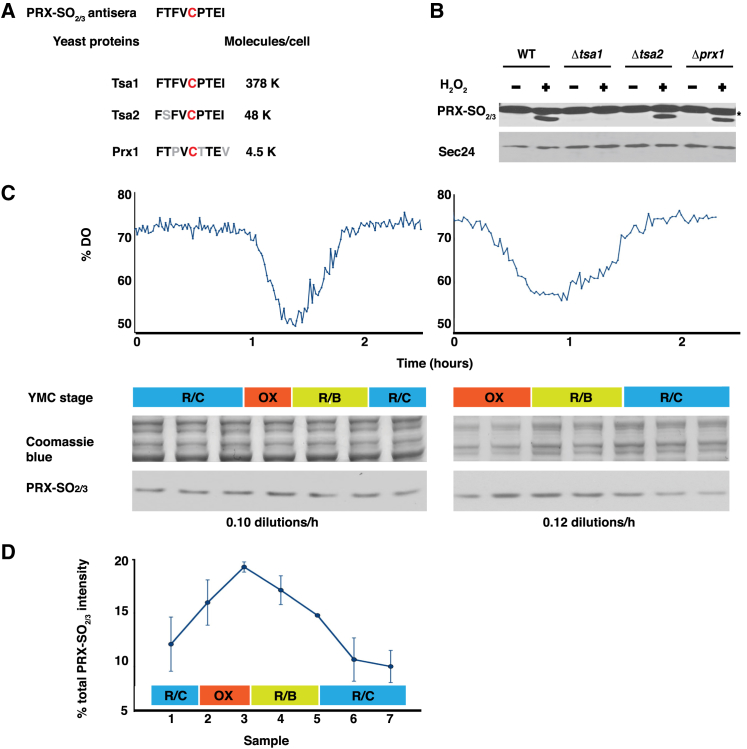
PRX Tsa1 Undergoes Cycles of Oxidation across the YRO (A) Three PRXs in the yeast *S. cerevisiae* have the nine amino acid sequence recognized by the PRX-SO_2/3_ antisera when over-oxidized. (B) Western blot showing that over-oxidized Tsa1 is recognized by the PRX-SO_2/3_ antisera in a redox-dependent manner. Samples were harvested immediately before (−) or 15 min after (+) the addition of hydrogen peroxide (1 mM final concentration). The asterisk marks a cross-reacting band. (C) YROs were obtained using 0.1 or 0.12 dilutions/hr (sampled every 20 or 12 min, respectively) and monitored using the dissolved oxygen trace (top). Bottom: whole-cell extracts obtained from samples taken across the oscillation were analyzed for PRX over-oxidation by western blotting. (D) Grouped data showing the normalized PRX-SO_2/3_ intensity over the YRO, peaking around late OX phase (mean ± SEM, n = 3, p = 0.024 for time effect by two-way ANOVA).

Peroxiredoxins (PRXs) are abundant thiol-specific cellular peroxidases that employ a conserved cysteine residue for the reduction of intracellular peroxides. Oxidized PRX usually dimerizes via a disulphide and is re-reduced by the thioredoxin system or may become over-oxidized to the sulphinic form (SO_2_) and subsequently recycled by sulphiredoxin (Srx) [25]. These ubiquitous antioxidants were recently suggested to constitute universal markers for circadian rhythms, as they exhibit a ∼24-hr rhythm in cysteine over-oxidation that persists (albeit perturbed) in circadian clock mutants and also in the absence of nascent gene expression, e.g., in mammalian erythrocytes [8, 26]. Although PRX over-oxidation cycles are thought to reflect an underlying oscillation in cytosolic redox balance, it is unclear whether PRX activity is required for clock function. The YRO coordinates with many cellular processes (Table S1), including mitochondrial and cytosolic redox metabolism. We therefore postulated that if PRX over-oxidation reflects a rhythm in the production of reactive oxygen species (ROS) and/or reducing equivalents, PRX oxidation should also be driven by the YRO. *S. cerevisiae* expresses three PRXs that contain the conserved 9-mer motif recognized by commercial antisera: Tsa1, Tsa2, and mitochondrial Prx1 (Figure 3A). By using single gene deletions and peroxide treatment, we observed oxidation-specific bands at the expected molecular weight (∼22 kD) only in strains with wild-type *TSA1*, indicating that the anti-PRX-SO_2/3_ antiserum specifically recognizes over-oxidized Tsa1 (Figure 3B). Samples were then collected over the course of the YRO, run at two different dilution rates in three independent experiments, to test whether PRX over-oxidation correlated with YROs of different period, and analyzed by western blotting. The PRX-SO_2/3_ signal showed a fixed-phase relationship with the YRO, consistently peaking just after the end of oxidative phase (OX, Figure 1D), when ROS generated as respiratory by-products are likely to be maximal (Figures 3C and 3D).

### PRX Activity Affects, but Is Not Required for, Ultradian or Circadian Rhythms

**Figure 4:**
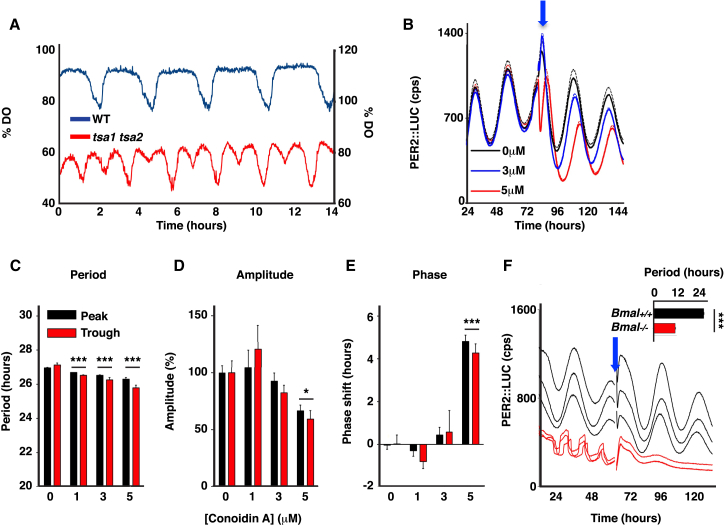
PRXs Contribute to YROs but Are Not Required for Cycling (A) Dissolved oxygen trace for a wild-type strain (period 2.77 hr, SD 0.26, n = 17) and a *tsa1 tsa2* double mutant strain (period 2.37 hr, SD 0.18, n = 6, p < 0.001). (B) Grouped detrended bioluminescence traces (mean ± SEM, n = 3) showing the effect of a 2-cys PRX inhibitior on PER2::LUCIFERASE rhythms in immortalized mouse fibroblasts added at the peak or trough (data not shown). The blue arrow indicates the point at which conoidin A or vehicle was added. (C–E) Conoidin A subtly shortens circadian period (p < 0.0001; C) and has robust effects upon amplitude (p = 0.0024; D) and circadian phase (p < 0.0001; E). Mean ± SEM is shown for (C)–(E) (n = 3, p values are two-way ANOVA, concentration effect). Asterisks report Bonferroni post-test p values for each drug concentration versus vehicle control. (F) Immortalized *Bmal1*^*−/−*^ fibroblasts exhibit an ultradian rhythm of PER2::LUCIFERASE activity under certain culture conditions, abolished following a complete media change. Inset shows mean period ± SEM before the media change (unpaired t test versus wild-type controls, p < 0.0001, n = 16).

To test whether PRX activity is necessary for the YRO, we knocked out the major cytosolic PRXs, *TSA1* and *TSA2*, and also *PRX1*. Since *TSA1* and *TSA2* act cooperatively and/or semi-redundantly, we also tested a *tsa1 tsa2* strain and an *srx1* strain in order to establish whether catalytic cycling of PRX over-oxidation might be important. All of the strains tested underwent respiratory oscillations, showing that PRX activity is not required for cycling (Figures 4 and S3B–S3F). However, Tsa1 and Tsa2 together make some contribution to the integrity of this temporal metabolic program because the double deletion strain has a distinct dissolved oxygen profile, with a pronounced “dip” in the trace during the reductive phase and cycles with a slightly shorter period (Figure 4A). This dip may reflect a transient respiratory burst during the “reductive/charging” (R/C) portion of the cycle, suggesting that one function of Tsa1/2 may be to stably maintain reductive metabolism. Catalytic recycling of Tsa1/2 by Srx1 makes no YRO contribution, as the *srx1* strain is phenotypically similar to wild-type (Figures S3E and S3F). Consistent with this result, circadian PRX over-oxidation cycles persist in red blood cells lacking Srx [27] but require proteasomal activity [27], and we speculate PRX-SO_2/3_ may similarly be degraded by the 20S proteasome. We also note that a strain lacking Prx1 exhibits a lengthened period (Figure S3F), suggesting that antioxidant mitochondrial balance also contributes to this respiratory oscillation, as might be expected for an oscillation with a redox cycle at its core [13].

The equivalent PRX loss-of-function experiment in mammalian cells is impractical as there are six PRX isoforms, so we used conoidin A (CA), a naturally occurring, membrane-permeable irreversible (2-cys) PRX inhibitor that reacts with the catalytic (peroxidatic) cysteine residue [28]. We confirmed CA activity by observing that it dose dependently blocks over-oxidation of cellular PRX in mouse fibroblasts following 30-min treatment with 2 mM H_2_O_2_ (Figure S3). Circadian bioluminescence assays revealed that at sub-toxic concentrations (≤5 μM), PRX inactivation subtly, but significantly, shortened the period of oscillation, accompanied by much larger dose-dependent effects on the amplitude and phase of PERIOD2::LUCIFERASE rhythms (Figures 4B–4E). This result echoes previous observations of circadian activity in 2-cys PRX null mutants in *Synechococcus elongatus* (a cyanobacterium) and *Arabidopsis thaliana* (a plant) [8]. At higher CA concentrations (>5 μM), cells were dead within 24 hr. These results suggest that PRX activity modulates, but is not required for, cellular circadian rhythmicity. These results are similar to those observed for YROs.

### Comparative Chronobiology Offers New Insights toward Mechanism

Collectively, our data show that conserved post-translational features of circadian rhythms in eukaryotes are also a feature of YROs in an organism not known for 24-hr periodicity and that YROs are not merely a function of the CDC. In our view, these results are consistent with two different models. (1) There are a number of conserved cellular mechanisms involved in numerous cellular functions that are also permissive for biological oscillations in the frequency range of hours. These enzymes do not have a specific role in the circadian clock but have a more general cellular function. (2) Yeast respiratory oscillations rely upon the same central timekeeping mechanisms employed by circadian clocks in higher organisms, and this reflects a common origin.

In the first model, recruitment of the same post-translational mechanisms to sustain biological rhythms of quite different periods means that they are the chronobiological equivalent of housekeeping enzymes. This could explain why, for example, CK1 activity also determines the period of circatidal rhythms in *Eurydice pulchra*, which is driven by a non-circadian clock [29]. The second model would be supported if *S. cerevisiae* evolved under conditions where selection favored rapid growth over circadian timing. Budding yeast is known to have undergone genome duplications followed by massive gene loss that would have facilitated this process [30]. The benefits conferred by clock-controlled temporal segregation of metabolism and gene expression would continue to bestow a fitness advantage, with the consequence that the period of the oscillation shortened to allow a faster cell cycle. In support of the possibility that a circadian clock became the YRO through gene deletion, we were interested to observe that under certain conditions, fibroblasts homozygous null for the “core clock gene” Bmal1 exhibit a pronounced ultradian rhythm in “clock gene” expression (Figure 4F), echoing previous observations made using pacemaker neurons from Bmal1 null mice [31].

In either case, by comparing similar biological rhythms with different periods in very distantly related eukaryotes (yeast versus mouse), our approach offers the potential to identify the processes that determine the speed at which biological clocks run. The Last Eukaryotic Common Ancestor (LECA) possessed mitochondria, a nucleus, metabolic pathways that included glycolysis, and the pentose phosphate pathway, as well as a cell cycle regulated by cyclins and cyclin-dependent kinases [32, 33]. We speculate that it also had a circadian clock.

## Experimental Procedures

### Strain Construction and Growth Curves in Yeast

Yeast deletion strains were made by insertion of the KanMX and/or NatMX cassette in the CEN.PK113-7D background [34] using standard genetic methods (Table S3).

The optical density (OD) of wild-type and *swe1* strains was measured over time, and a corrected value was obtained by subtracting background density (OD of media at each time point). Relative growth rates were determined by linear regression during log phase growth in batch cultures. Cell number was counted using a haemocytometer, and turbidity measurements were obtained using a Bioscreen C machine (Lab Systems). These experiments were carried out at 30°C using the same media as that used to feed the bioreactor.

### Protein Preparation and Detection

Yeast whole-cell extracts were prepared by trichloroacetic acid (TCA) precipitation as described [35], with the addition of the following protease inhibitors (Sigma): aminobenzamide dihydrochloride (200 μg/ml), antipain (1 μg/ml), aprotinin (1 μg/ml), leupeptin (1 μg/ml), chymostatin (1 μg/ml), PMSF (200 μg/ml), TPCK (50 μg/ml), and pepstatin (1 μg/ml). Gel electrophoresis and western blotting were carried out as described [8], except that mini NuPAGE gels (Life Technologies) were used and proteins were wet transferred to PVDF. PRX-SO_2/3_ antibody (ab16830) was purchased from Abcam.

### YROs

Respiratory oscillations were generated as described [10, 36], using a 7.5 L New Brunswick Celligen 115/Bioreactor containing 2 L media at pH 3.4 at 30°C with 4 L/min aeration, 550 rpm agitation. The pH was maintained with 10% NaOH. Unless otherwise stated, all experiments used a continuous flow rate of 0.1 dilutions/hr. The oxygen probe was calibrated prior to each experiment. All experiments on mutant strains were conducted at least twice, while those involving dose response curves represent the combined results from two (LH846) or three (PF670462) experiments. PF670462 and LH846 were purchased from Tocris Bioscience.

### Flow Cytometry

DNA was stained using propidium iodide (PI) using standard methods. Flow cytometry was carried out using a FACSCalibur flow cytometer (Becton Dickinson). 20,000 cells were scored for each sample.

### Data Analysis

Period length was calculated using a custom-built script in MATLAB. To automatically identify minima, we used a sliding window smoothing algorithm on the dissolved oxygen data to reduce measurement noise. All local minima points were then identified in the smoothed data. A period was defined between each pair of adjacent minima points. t tests were carried out in Excel, using the T.TEST function for two-tailed samples of unequal variance. NIH ImageJ software was used to quantify the intensity of bands on western blots and protein gels. Other statistical analyses were performed using Graphpad Prism. Intensities from western blotting were corrected for variations in total protein concentration in each lane and standardized based on total signal intensity across the oscillation.

### Culture and Manipulation of Mammalian Cells

Primary fibroblasts homozygous for PERIOD2::LUCIFERASE [37] were isolated from the lung tissue of adult males and cultured as described previously [38] and then immortalized by serial passage [39]. GSK3β^−/−^ and wild-type control mouse embryonic fibroblasts from [40] were stably transfected with a plasmid encoding *Per2:luc*. Passage number did not exceed 20. All animal work was licensed under the UK Animals (Scientific Procedures) Act of 1986 with local ethical approval. Cell lysis and immunoblotting is described in the legend for Figure S3.

### Monitoring of Circadian Rhythms

Cells were seeded at a density of 10^5^ per 35-mm dish and grown to complete confluence with regular media changes as described previously [38]. Bioluminescence assays were performed in HEPES-buffered “Air Medium” [38] supplemented with 10% HyClone FetalClone III serum, 1 mM luciferin, 2% B-27 supplement, and 1× Glutamax in all cases, with the exception that 1% serum was used in the recording from Bmal1^−/−^ fibroblasts and wild-type controls, and these cells were cultured under 12 hr:12 hr 32°C:37°C temperature cycles for 2 weeks prior to changing to Air Medium for the recording at constant 37°C. Drugs were purchased from Cayman Chemical, dissolved in DMSO, and then diluted into Air Medium such that the final concentration of DMSO did not exceed 0.1%. Within each experiment, DMSO concentration was internally controlled (i.e., equal DMSO at 0 μM and 1 μM drug). Mammalian bioluminescence experiments were performed using a Lumicycle (Actimetrics) at constant 37°C. Lumicycle data were detrended to remove baseline changes and then fit with a damped sine wave in order to determine circadian period, amplitude and phase as in [22]. This and all other statistical analyses were performed using Graphpad Prism.

## Author Contributions

H.C.C. and J.S.O. designed the study and wrote the paper. H.C.C. and C.A.Z. generated data in yeast. J.S.O. and K.A.F. performed experiments in mammalian cells.

## Supplemental Information

Document S1. Figures S1–S3 and Tables S1–S3

Document S2. Article plus Supplemental Information
