# Supplementary material for: Metabolic Cycles in Yeast Share Features Conserved among Circadian Rhythms
Source: Curr Biol. 2015 Apr 20;25(8):1056–62. doi: 10.1016/j.cub.2015.02.035 (PMC4406945; doi:10.1016/j.cub.2015.02.035)
Supplement: Document S1. Figures S1–S3 and Tables S1–S3 [file mmc1.pdf]

Current Biology
Supplemental Information

# Metabolic Cycles in Yeast
# Share Features Conserved among Circadian Rhythms

Helen C. Causton, Kevin A. Feeney, Christine A. Ziegler, and John O'Neill

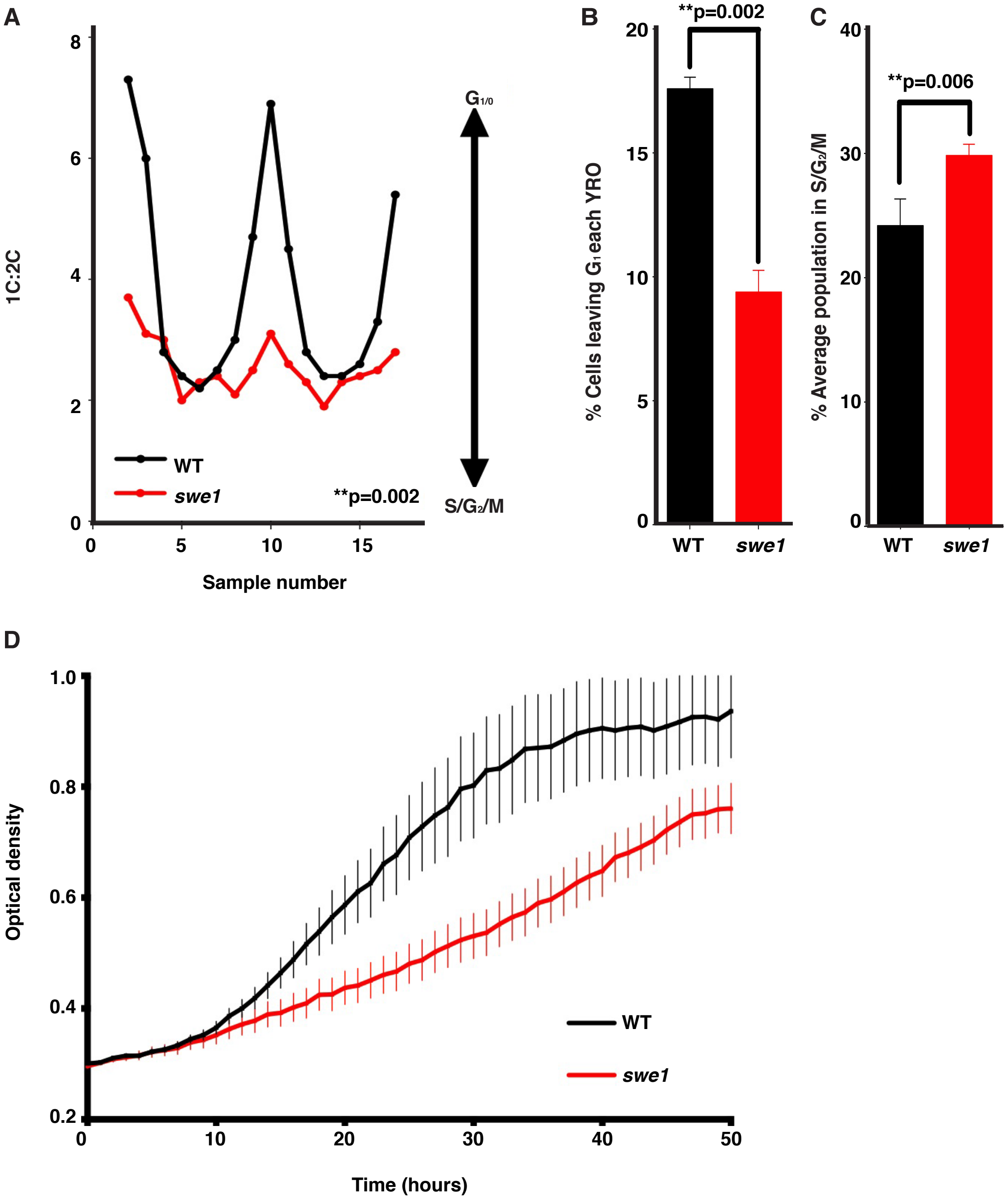


**Figure S1, relating to Figure 1. Strains deleted for *SWE1* show less variation in DNA content across the YRO, leave $G_1$ less frequently and grow slower than wild type under YRO/bioreactor and standard growth conditions** (**A**) Samples harvested across two oscillations were analysed for DNA content by propidium iodide staining and FACs analysis. Paired t-test, p=0.002. (**B**) Bar graph showing fewer *swe1* cells leave $G_1$ per respiratory oscillation when growing in the bioreactor. Paired t-test, p=0.002. (**C**) Bar graph showing that, on average, more cells are in the S/G2 or M phases of the cell cycle in *swe1* strains than in wild type under YRO/bioreactor conditions. Paired t-test, p=0.006. (**D**) Growth curves for wild type and *swe1* strains under standard growth conditions. Mean±SEM, n=4; 2-way ANOVA, p<0.0001 for time vs. genotype interaction.

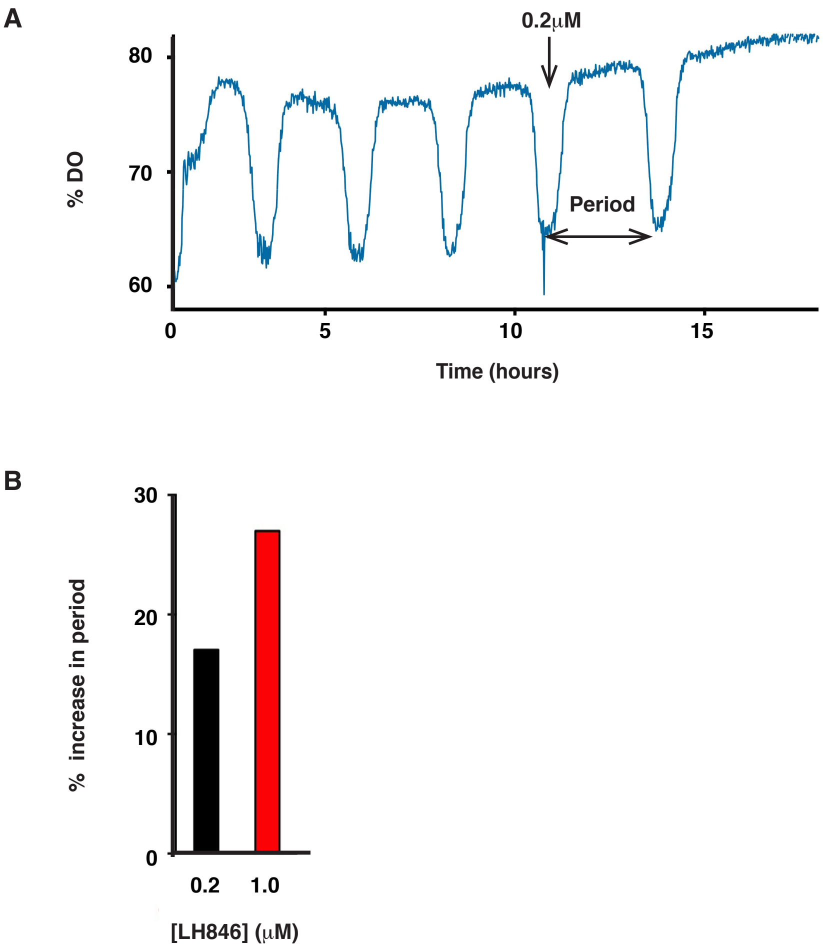


**Figure S2, relating to Figure 2. Casein kinase inhibitor LH846 has a dose-dependent effect on the period of the YRO.** (**A**) Representative dissolved oxygen trace indicating the time of addition of LH846. (**B**) Bar graph showing increase in period of oscillation in the presence of drug. Results from two independent experiments.

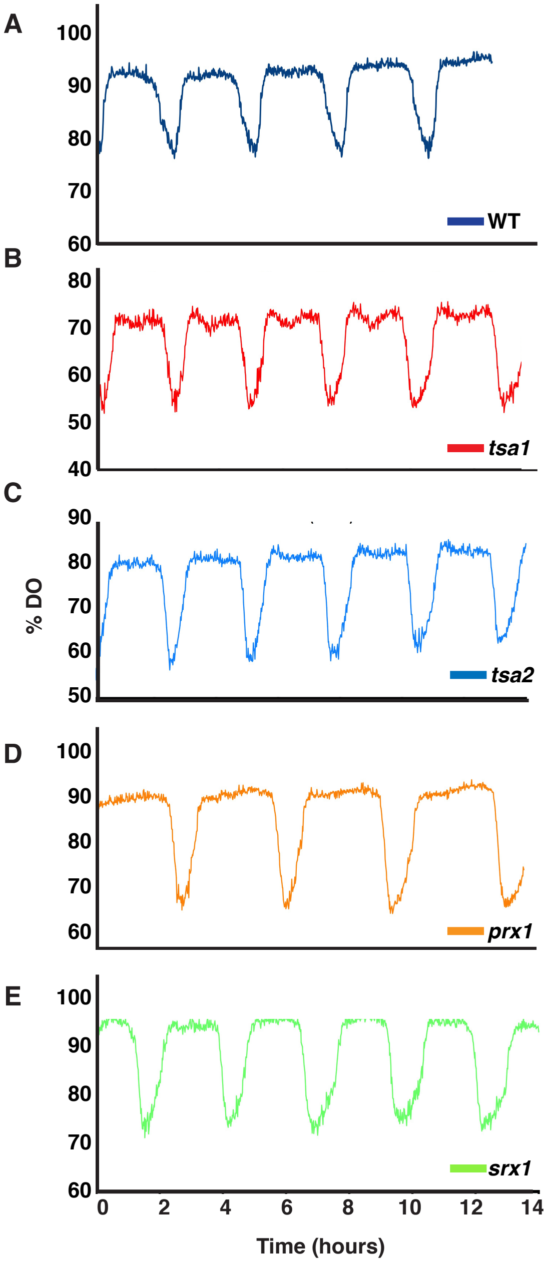


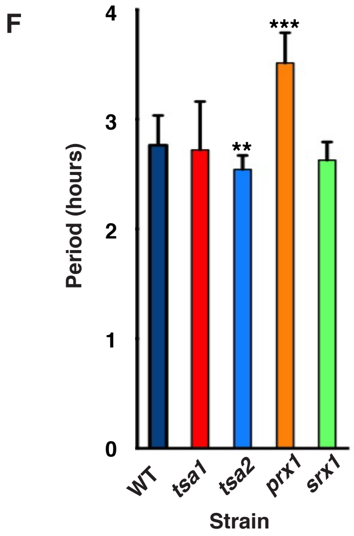


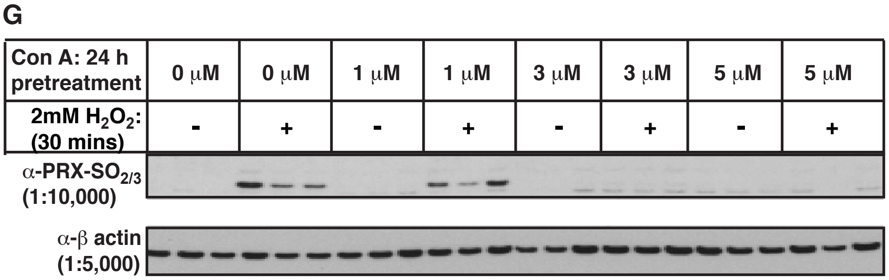


**Figure S3, relating to Figure 4. Individual peroxiredoxins are not required for YROs and Conoidin A acts to block over-oxidation of cellular PRX in mouse fibroblasts treated with peroxide.** (**A-E**) Representative dissolved oxygen trace for wild type and mutant strains. (**F**) Periods are wild type 2.77 h, SD 0.26, n=17; *tsa1* 2.72h, SD 0.44, n= 7, p=0.807; *tsa2* 2.53h, SD 0.13, n= 9, p=0.009; *prx1* 3.50h, SD 0.27, n= 5, p=0.001; *srx1* 2.62h, SD 0.17, n= 6, p=0.162. Mean ± SEM, p-values are t-test vs. wild type. (**G**) Western blot showing dose-dependent inhibition of PRX over-oxidation by 30 minutes 2 mM $H_2O_2$ treatment, following 24-hour pre-treatment with varying concentrations of conoidin A. SDS-PAGE was performed under reducing conditions, and performed as [S16] except that cells were pre-treated with 20 mM NEM in ice cold PBS for 15 minutes prior to lysis to alkylate any free thiol groups. Three biological replicates are shown per condition.

| | Mammalian Cellular Circadian Rhythm | Yeast Respiratory Oscillation |
|---|---|---|
| **Cell autonomous, persists under constant conditions** | • Mouse fibroblasts and neurons [S1-S3]. | • Period is consistent within a given strain/set of conditions [S4, S5-S10]. |
| **Temperature compensated** | • In mouse fibroblasts [S11, S12]. | • In *Schizosaccharomyces pombe* and *Saccharomyces cerevisiae* [S7, S13, S14]. |
| **Involves redox/metabolic cycles** | • In human & mouse erythrocytes [S15, S16], mouse fibroblasts [S17, S18] and mouse myoblasts [S19].<br>• Mitochondrial metabolism [S19].<br>• Glucose utilization [S20]. | • Redox balance<br>NAD(P)H [S6, S8, S21, S22-S25].<br>Glutathione and GSH [S5, S21, S24]<br>• Mitochondrial metabolism [S6, S23, S26, S27].<br>• Glucose utilization [S8, S9, S28]. |
| **Involves rhythmic transcription and chromatin modification** | • In mouse fibroblasts [S29, S30-S32]. | • Transcription [S6, S9, S33, S34].<br>• Histone acetylation [S35].<br>• Chromatin modification [S36]. |
| **Involves temporal regulation of haem and carbon monoxide** | • Agonists of clock gene REV-ERB's haem binding site resets clock in mouse fibroblasts [S37].<br>• Haem synthesis is circadian regulated [S38].<br>• Other clock genes reported as functionally regulated by haem [S39, S40]. | • The concentration of aminolevulinic acid, a rate-limiting metabolite in the synthesis of haem, oscillates [S24].<br>• Addition of CO can induce phase advancement, strains deleted for haem oxygenase oscillate with a longer period [S41]. |
| **Coupled with DNA replication and cell division** | • In mouse fibroblasts [S2, S42-S44]. | • DNA replication is gated to the reductive phase of the cycle [S6, S9, S33]. This may function to reduce the mutation rate [S7, S45].<br>• Oscillations are typically sychronised with the budding index [S25, S46, S47].<br>• Respiratory oscillations can occur without cell division cycling [S48].<br>• Swe1 is required for entry to the cell cycle after $G_1$ arrest [S49] and *SWE1* expression varies more than 10 fold across the YRO [S6]. |

**Table S1.**
**Note that the mammalian references pertain specifically to observations made using cultured mammalian cells *in vitro* so as to be comparable with the yeast references.**

| | | Casein kinase 1 (CK1) | Glycogen synthase kinase 3 (GSK3) |
|---|---|---|---|
| | Description | • Family of nucleocytoplasmic Ser/Thr kinases<br>• Highly conserved in eukaryotes, ubiquitously expressed<br>• Auto-phosphorylation inactivates<br>• Usually requires a priming phosphate (pS/T-X-X-S)<br>• Can act synergistically with other kinases, such as GSK3 and PKA, at multisite phosphorylation domains to regulate protein stability and nucleo-cytoplasmic shuttling [S50, S51]. | • Family of nucleocytoplasmic Ser/Thr kinases<br>• Highly conserved in eukaryotes, ubiquitously expressed<br>• Akt/PKB phosphorylation inactivates<br>• Usually requires a priming phosphate (S/T-X-X-X-pS)<br>• Can act synergistically with other kinases, such as CK1 and CK2, at multisite phosphorylation domains to regulate protein stability and nucleo-cytoplasmic shuttling [S50, S52]. |
| **Cellular functions (non-circadian)** | Mammals | • 5 isoforms (7 genes) in mammals (α, β, γ1-3, δ, ε) with multiple cellular substrates and partners.<br>• Important roles in membrane trafficking, DNA replication/repair, cytokinesis, vesicular transport, ribosome biogenesis, and transcription [S53, S54].<br>• Several CK1 isoforms work co-ordinately regulate wnt signalling [S55].<br>• Targets proteins such as β-catenin and IκB for ubiquitin-mediated proteasomal degradation through recruitment of F-box proteins (e.g. β-TRCP) that recognise the phosphodegron (Dp-S-G-X-XpS) [S56]. | • 2 isoforms in mammals (α/β) with multiple cellular substrates and partners [S52].<br>• Phosphorylates enzymes such as glycogen synthase and IRS1 to regulate glucose homeostasis [S57, S58].<br>• Essential role in development (part of canonical β-catenin/wnt signalling pathway) [S59].<br>• Regulates apoptosis, cell proliferation and migration [S60].<br>• Important signalling role in both the adaptive and innate immune responses [S61].<br>• Regulates cellular response to DNA damage e.g. through phosphorylation of p53 [S62]. |
| **Cellular functions (non-circadian)** | Flies | • 8 family members with diverse roles in cytoskeletal polarisation/morphogenesis [S63], glial cell migration [S64], olfactory learning [S65], sperm individualization [S66], regulator of Wnt and Hedgehog signaling [S67].<br>• Synergystic regulation of β-catenin/Armadillo with GSK3, similarly to mammalian cells [S68]. | • Fly ortholog (*Shaggy, Sgg*) is a segment polarity gene best known as a repressor of Wingless (Wg) signalling [S69], but also required for normal growth of larval and imaginal tissues [S70].<br>• Also involved in attachment of the mitotic spindle at the cell cortex [S71], and repression of Hedgehog signaling where it acts synergistically with CK1 to regulate protein degradation [S72]. |
| **Cellular functions (non-circadian)** | Plants | • Plants encode dozens of CK1 homologs, mostly uncharacterised. Roles include subcellular targeting [S73], root development, plant hormone sensitivity [S74], microtubule organization [S75] and starch metabolism [S76]. | • The large family of GSK3 homologs in plants are implicated in diverse roles such as regulation of vascular development [S77], abscisic acid signalling [S78], floral development [S79], cell growth/differentiation [S80].<br>• GSK3-like kinase BIN3 phosphorylates transcription factor BIN2, to stabilise it and thereby negatively regulate brassinosteroid signalling [S81]. |
| **Cellular functions (non-circadian)** | Fungi (*S. cerevisiae*) | • 4 CK1 isoforms in *S. cerevisae*: – Yck1/2 functions in ROS signalling, to directly integrate signals from oxygen and glucose and repress transcription [S82].<br>• CK1δ homolog, Hrr25 has multiple functions including repression of calcineurin signalling [S83]. | • Yeast homolog, Rim11, part of a feed forward loop that switches diploid yeast from a proliferative cycle to the meiotic cycle in response to nutrients [S84].<br>• Acts with PKA to regulate the stability of the stress-responsive transcription factor Cin5 [S85]. |
| **Circadian clock function** | Mammals | • The gain-of-function tau mutation in CK1ε shortens circadian period by ~3 hours in homozygotes [S86-S88].<br>• Human familial sleep disorders (early awakening) segregate with mutations in human CK1δ or hPER2 phosphorylation sites [S89, S90].<br>• CK1δ/ε determine PER1/2 protein degradation, complex formation and nuclear import/export in the absence of both enzymes, the canonical transcriptional oscillator stops completely [S91, S92]. CK1α also recently implicated [S93]. | • GSK3 exhibits a cell-autonomous phosphorylation rhythm [S94, S95].<br>• Knockdown/pharmacological inhibition of GSK3β shortens circadian period [S96]. Constitutive activation lengthens circadian period [S94].<br>• Phosphorylates clock proteins BMAL1, CLOCK, CRY2, PER2 and REV-ERBα to regulate their stability [S97, S98, S99]. |
| **Circadian clock function** | Flies | • Early mutagenesis screens identified CK1δ/ε homolog (*doubletime, dbt*), which regulates period length through phosphorylation of dPER [S100].<br>• Phosphorylation determines dPER turnover kinetics, complex formation and nucleocytoplasmic shuttling [S100, S101]. | • GSK3β homolog (*shaggy, sgg*) was originally implicated in timekeeping in Drosophila mutants that exhibit altered circadian period [S102].<br>• GSK3 phosphorylates components of the core clock machinery (*e.g.*, TIMELESS) to regulate clock complex stability, localisation and periodicity [S103]. |
| **Circadian clock function** | Plants/ algae | • CK1 over-expression or inhibition lengthens circadian period in *O. tauri* ([S104, S105].<br>• CK1 homologs phosphorylate Cryptochrome (blue light sensor) in *O. tauri* and *A. thaliana* [S105, S106]. | • Pharmacological inhibitors of GSK3 shorten circadian period dose-dependently in *O. tauri* [S104].<br>• Clock-relevant targets are not known at present. |
| **Circadian clock function** | Fungi (*N. Crassa*) | • A CK1δ/ε homolog phosphorylates FRQ and WC1/2 to regulate the kinetics of protein turnover, thereby determining circadian period length [S107, S108, S109]. | • A GSK3 homolog phosphorylates WC-1/2 to regulate protein abundance, with knockdown leading to WC-1 accumulation and shortened circadian period [S110]. |

**Table S2. A comparison between the cellular and circadian functions of CK1 and GSK3 in model eukaryotes.**

All the strains are isogenic and were made in the prototrophic CEN.PK background [S111].

| | |
|---|---|
| HCY1377 | *Mata TSA1::KanMX* |
| HCY1381 | *Mata TSA2::KanMX* |
| HCY1385 | *Mata PRX1::KanMX* |
| HCY1414 | *Mata SRX1::KanMX* |
| HCY1427 | *Mata TSA1::NatMX TSA2::KanMX* |
| HCY1445 | *Mata RIM11::KanMX* |
| HCY1465 | *Mata SWE1::KanMX* |

**Table S3. Strains and genotypes**

Supplemental References


S1. Welsh, D.K., Yoo, S.H., Liu, A.C., Takahashi, J.S., and Kay, S.A. (2004). Bioluminescence imaging of individual fibroblasts reveals persistent, independently phased circadian rhythms of clock gene expression. Curr Biol *14*, 2289-2295.

S2. Nagoshi, E., Saini, C., Bauer, C., Laroche, T., Naef, F., and Schibler, U. (2004). Circadian gene expression in individual fibroblasts: cell-autonomous and self-sustained oscillators pass time to daughter cells. Cell *119*, 693-705.

S3. Welsh, D.K., Logothetis, D.E., Meister, M., and Reppert, S.M. (1995). Individual neurons dissociated from rat suprachiasmatic nucleus express independently phased circadian firing rhythms. Neuron *14*, 697-706.

S4. Keulers, M., Satroutdinov, A.D., Suzuki, T., and Kuriyama, H. (1996). Synchronization affector of autonomous short-period-sustained oscillation of Saccharomyces cerevisiae. Yeast *12*, 673-682.

S5. Sohn, H.-Y., and Kuriyama, H. (2001). Ultradian metabolic oscillation of *Saccharomyces cerevisiae* during aerobic continuous culture: hydrogen sulphide, a population synchronizer, is produced by sulphite reductase. Yeast *18*, 125-135.

S6. Tu, B.P., Kudlicki, A., Rowicka, M., and McKnight, S.L. (2005). Logic of the yeast metabolic cycle: temporal compartmentalization of cellular processes. Science *310*, 1152-1158.

S7. Chen, Z., and McKnight, S.L. (2007). A conserved DNA damage response pathway responsible for coupling the cell division cycle to the circadian and metabolic cycles. Cell Cycle *6*, 2906-2912.

S8. Ouyang, Y., Xu, Q., Mitsui, K., Motizuki, M., and Xu, Z. (2011). PSK2 coordinates glucose metabolism and utilization to maintain ultradian clock-coupled respiratory oscillation in Saccharomyces cerevisiae yeast. Archives of biochemistry and biophysics *509*, 52-58.

S9. Chin, S.L., Marcus, I.M., Klevecz, R.R., and Li, C.M. (2012). Dynamics of oscillatory phenotypes in Saccharomyces cerevisiae reveal a network of genome-wide transcriptional oscillators. The FEBS journal *279*, 1119-1130.

S10. Eelderink-Chen, Z., Mazzotta, G., Sturre, M., Bosman, J., Roenneberg, T., and Merrow, M. (2010). A circadian clock in Saccharomyces cerevisiae. Proc Natl Acad Sci U S A *107*, 2043-2047.

S11. Tsuchiya, Y., Akashi, M., and Nishida, E. (2003). Temperature compensation and temperature resetting of circadian rhythms in mammalian cultured fibroblasts. Genes to cells : devoted to molecular & cellular mechanisms *8*, 713-720.

S12. Izumo, M., Johnson, C.H., and Yamazaki, S. (2003). Circadian gene expression in mammalian fibroblasts revealed by real-time luminescence reporting: temperature compensation and damping. Proc Natl Acad Sci U S A *100*, 16089-16094.

S13. Kippert, F., and Lloyd, D. (1995). A temperature-compensated ultradian clock ticks in Schizosaccharomyces pombe. Microbiology *141 ( Pt 4)*, 883-890.

S14. Murray, D.B., Roller, S., Kuriyama, H., and Lloyd, D. (2001). Clock control of ultradian respiratory oscillation found during yeast continuous culture. Journal of bacteriology *183*, 7253-7259.

S15. Cho, C.S., Yoon, H.J., Kim, J.Y., Woo, H.A., and Rhee, S.G. (2014). Circadian rhythm of hyperoxidized peroxiredoxin II is determined by hemoglobin autoxidation and the 20S proteasome in red blood cells. Proc Natl Acad Sci U S A.

S16. Milev, N.B., Rey, G., Valekunja, U.K., Edgar, R.S., O'Neill, J.S., and Reddy, A.B. (2014). Analysis of the Redox Oscillations in the Circadian Clockwork. Methods in Enzymology *In press*, 617-642.

S17. O'Neill, J.S., and Reddy, A.B. (2011). Circadian clocks in human red blood cells. Nature *469*, 498-503.

S18. O'Neill, J.S., and Feeney, K.A. (2013). Circadian Redox and Metabolic Oscillations in Mammalian Systems. Antioxidants & redox signaling.

S19. Peek, C.B., Affinati, A.H., Ramsey, K.M., Kuo, H.Y., Yu, W., Sena, L.A., Ilkayeva, O., Marcheva, B., Kobayashi, Y., Omura, C., et al. (2013). Circadian clock NAD+ cycle drives mitochondrial oxidative metabolism in mice. Science *342*, 1243417.

S20. Paulose, J.K., Rucker, E.B., 3rd, and Cassone, V.M. (2012). Toward the beginning of time: circadian rhythms in metabolism precede rhythms in clock gene expression in mouse embryonic stem cells. PLoS One *7*, e49555.

S21. Murray, D.B., Engelen, F., Lloyd, D., and Kuriyama, H. (1999). Involvement of glutathione in the regulation of respiratory oscillation during a continuous culture of *Saccharomyces cerevisiae*. Microbiol. *145*, 2739-2745.

S22. Wang, J., Liu, W., Uno, T., Tonozuka, H., Mitsui, K., and Tsurugi, K. (2000). Cellular stress responses oscillate in synchronization with the ultradian oscillation of energy metabolism in the yeast *Saccharomyces cerevisiae*. FEMS Microbiol. Lett. *189*, 9-13.

S23. Lloyd, D., Salgado, L.E., Turner, M.P., Suller, M.T., and Murray, D. (2002). Cycles of mitochondrial energization driven by the ultradian clock in a continuous culture of Saccharomyces cerevisiae. Microbiology *148*, 3715-3724.

S24. Tu, B.P., Mohler, R.E., Liu, J.C., Dombek, K.M., Young, E.T., Synovec, R.E., and McKnight, S.L. (2007). Cyclic changes in metabolic state during the life of a yeast cell. P.N.A.S (USA) *104*, 16886-16891.

S25. Xu, Z., and Tsurugi, K. (2007). Destabilization of energy-metabolism oscillation in the absence of trehalose synthesis in the chemostat culture of yeast. Archives of biochemistry and biophysics *464*, 350-358.

S26. Kwak, W.J., Kwon, G.S., Jin, I., Kuriyama, H., and Sohn, H.Y. (2003). Involvement of oxidative stress in the regulation of H(2)S production during ultradian metabolic oscillation of Saccharomyces cerevisiae. FEMS Microbiol Lett *219*, 99-104.

S27. Sohn, H.-Y., Kum, E.-J., Kwon, G.-S., Jin, I., and Kuriyama, H. (2005). Regulation of branched-chain, and sulfur-containing amino acid metabolism by glutathione during ultradian metabolic oscillation of *Saccharomyces cerevisiae*. J. Microbiol. *43*, 375-380.

S28. Wittmann, C., Hans, M., van Winden, W.A., Ras, C., and Heijnen, J.J. (2005). Dynamics of intracellular metabolites of glycolysis and TCA cycle during cell-cycle-related oscillation in Saccharomyces cerevisiae. Biotechnology and bioengineering *89*, 839-847.

S29. Naruse, Y., Oh-hashi, K., Iijima, N., Naruse, M., Yoshioka, H., and Tanaka, M. (2004). Circadian and light-induced transcription of clock gene Per1 depends on histone acetylation and deacetylation. Mol Cell Biol *24*, 6278-6287.

S30. Fogg, P.C., O'Neill, J.S., Dobrzycki, T., Calvert, S., Lord, E.C., McIntosh, R.L., Elliott, C.J., Sweeney, S.T., Hastings, M.H., and Chawla, S. (2014). Class IIa histone deacetylases are conserved regulators of circadian function. J Biol Chem.

**S31.** **Nakahata, Y., Kaluzova, M., Grimaldi, B., Sahar, S., Hirayama, J., Chen, D., Guarente, L.P., and Sassone-Corsi, P. (2008). The NAD+-dependent deacetylase SIRT1 modulates CLOCK-mediated chromatin remodeling and circadian control. Cell *134*, 329-340.**

**S32.** **Aguilar-Arnal, L., Hakim, O., Patel, V.R., Baldi, P., Hager, G.L., and Sassone-Corsi, P. (2013). Cycles in spatial and temporal chromosomal organization driven by the circadian clock. Nature structural & molecular biology *20*, 1206-1213.**

**S33.** **Klevecz, R.R., Bolen, J., Forrest, G., and Murray, D.B. (2004). A genomewide oscillation in transcription gates DNA replication and cell cycle. P.N.A.S (USA) *101*, 1200-1205.**

**S34.** **Li, C.M., and Klevecz, R.R. (2006). A rapid genome-scale response of the transcriptional oscillator to perturbation reveals a period-doubling path to phenotypic change. Proc Natl Acad Sci U S A *103*, 16254-16259.**

**S35.** **Cai, L., Sutter, B.M., Li, B., and Tu, B.P. (2011). Acetyl-CoA induces cell growth and proliferation by promoting the acetylation of histones at growth genes. Mol Cell *42*, 426-437.**

**S36.** **Machné, R., and Murray, D.B. (2012). The yin and yang of yeast transcription: elements of a global feedback system between metabolism and chromatin. PLoS One *7*, e37906.**

**S37.** **Meng, Q.J., McMaster, A., Beesley, S., Lu, W.Q., Gibbs, J., Parks, D., Collins, J., Farrow, S., Donn, R., Ray, D., et al. (2008). Ligand modulation of REV-ERBalpha function resets the peripheral circadian clock in a phasic manner. J Cell Sci *121*, 3629-3635.**

**S38.** **Kaasik, K., and Lee, C.C. (2004). Reciprocal regulation of haem biosynthesis and the circadian clock in mammals. Nature *430*, 467-471.**

**S39.** **Dioum, E.M., Rutter, J., Tuckerman, J.R., Gonzalez, G., Gilles-Gonzalez, M.A., and McKnight, S.L. (2002). NPAS2: a gas-responsive transcription factor. Science *298*, 2385-2387.**

**S40.** **Yin, L., Wu, N., Curtin, J.C., Qatanani, M., Szwergold, N.R., Reid, R.A., Waitt, G.M., Parks, D.J., Pearce, K.H., Wisely, G.B., et al. (2007). Rev-erbalpha, a heme sensor that coordinates metabolic and circadian pathways. Science *318*, 1786-1789.**

**S41.** **Tu, B.P., and McKnight, S.L. (2009). Evidence of carbon monoxide-mediated phase advancement of the yeast metabolic cycle. Proc Natl Acad Sci U S A *106*, 14293-14296.**

**S42.** **Matsuo, T., Yamaguchi, S., Mitsui, S., Emi, A., Shimoda, F., and Okamura, H. (2003). Control mechanism of the circadian clock for timing of cell division in vivo. Science *302*, 255-259.**

**S43.** **Bieler, J., Cannavo, R., Gustafson, K., Gobet, C., Gatfield, D., and Naef, F. (2014). Robust synchronization of coupled circadian and cell cycle oscillators in single mammalian cells. Mol Syst Biol *10*, 739.**

**S44.** **Feillet, C., Krusche, P., Tamanini, F., Janssens, R.C., Downey, M.J., Martin, P., Teboul, M., Saito, S., Levi, F.A., Bretschneider, T., et al. (2014). Phase locking and multiple oscillating attractors for the coupled mammalian clock and cell cycle. Proc Natl Acad Sci U S A *111*, 9828-9833.**

**S45.** **Chen, Z., Odstrcil, E.A., Tu, B.P., and McKnight, S.L. (2007). Restriction of DNA replication to the reductive phase of the metabolic cycle protects genome integrity. Science *316*, 1916-1919.**

**S46.** **Porro, D., Martegani, E., Ranzi, B.M., and Alberghina, L. (1988). Oscillations in continuous cultures of budding yeast: a segregated parameter analysis. Biotechnology and bioengineering *32*, 411-417.**

**S47.** **Slavov, N., and Botstein, D. (2011). Coupling among growth rate response, metabolic cycle, and cell division cycle in yeast. Mol Biol Cell *22*, 1997-2009.**

S48. Slavov, N., Macinskas, J., Caudy, A., and Botstein, D. (2011). Metabolic cycling without cell division cycling in respiring yeast. Proc Natl Acad Sci U S A *108*, 19090-19095.
S49. Saracino, F., Bassler, J., Muzzini, D., Hurt, E., and Agostoni Carbone, M.L. (2004). The yeast kinase Swe1 is required for proper entry into cell cycle after arrest due to ribosome biogenesis and protein synthesis defects. Cell Cycle *3*, 648-654.
S50. Rena, G., Bain, J., Elliott, M., and Cohen, P. (2004). D4476, a cell-permeant inhibitor of CK1, suppresses the site-specific phosphorylation and nuclear exclusion of FOXO1a. EMBO reports *5*, 60-65.
S51. Hunter, T. (2007). The age of crosstalk: phosphorylation, ubiquitination, and beyond. Mol Cell *28*, 730-738.
S52. Xu, C., Kim, N.G., and Gumbiner, B.M. (2009). Regulation of protein stability by GSK3 mediated phosphorylation. Cell Cycle *8*, 4032-4039.
S53. Cheong, J.K., and Virshup, D.M. (2011). Casein kinase 1: Complexity in the family. The international journal of biochemistry & cell biology *43*, 465-469.
S54. Schittek, B., and Sinnberg, T. (2014). Biological functions of casein kinase 1 isoforms and putative roles in tumorigenesis. Molecular cancer *13*, 231.
S55. Del Valle-Perez, B., Arques, O., Vinyoles, M., de Herreros, A.G., and Dunach, M. (2011). Coordinated action of CK1 isoforms in canonical Wnt signaling. Mol Cell Biol *31*, 2877-2888.
S56. Zhao, B., Li, L., Tumaneng, K., Wang, C.Y., and Guan, K.L. (2010). A coordinated phosphorylation by Lats and CK1 regulates YAP stability through SCF(beta-TRCP). Genes Dev *24*, 72-85.
S57. Embi, N., Rylatt, D.B., and Cohen, P. (1980). Glycogen synthase kinase-3 from rabbit skeletal muscle. Separation from cyclic-AMP-dependent protein kinase and phosphorylase kinase. Eur J Biochem *107*, 519-527.
S58. Liberman, Z., and Eldar-Finkelman, H. (2005). Serine 332 phosphorylation of insulin receptor substrate-1 by glycogen synthase kinase-3 attenuates insulin signaling. J Biol Chem *280*, 4422-4428.
S59. Metcalfe, C., and Bienz, M. (2011). Inhibition of GSK3 by Wnt signalling--two contrasting models. J Cell Sci *124*, 3537-3544.
S60. Jope, R.S., and Johnson, G.V. (2004). The glamour and gloom of glycogen synthase kinase-3. Trends Biochem Sci *29*, 95-102.
S61. Wang, H., Brown, J., and Martin, M. (2011). Glycogen synthase kinase 3: a point of convergence for the host inflammatory response. Cytokine *53*, 130-140.
S62. Watcharasit, P., Bijur, G.N., Zmijewski, J.W., Song, L., Zmijewska, A., Chen, X., Johnson, G.V., and Jope, R.S. (2002). Direct, activating interaction between glycogen synthase kinase-3beta and p53 after DNA damage. Proc Natl Acad Sci U S A *99*, 7951-7955.
S63. Gault, W.J., Olguin, P., Weber, U., and Mlodzik, M. (2012). Drosophila CK1-gamma, gilgamesh, controls PCP-mediated morphogenesis through regulation of vesicle trafficking. J Cell Biol *196*, 605-621.
S64. Hummel, T., Attix, S., Gunning, D., and Zipursky, S.L. (2002). Temporal control of glial cell migration in the Drosophila eye requires gilgamesh, hedgehog, and eye specification genes. Neuron *33*, 193-203.
S65. Tan, Y., Yu, D., Pletting, J., and Davis, R.L. (2010). Gilgamesh is required for rutabaga-independent olfactory learning in Drosophila. Neuron *67*, 810-820.
S66. Nerusheva, O.O., Dorogova, N.V., Gubanova, N.V., Yudina, O.S., and Omelyanchuk, L.V. (2009). A GFP trap study uncovers the functions of Gilgamesh protein kinase in Drosophila melanogaster spermatogenesis. Cell biology international *33*, 586-593.

**S67. Price, M.A. (2006). CKI, there's more than one: casein kinase I family members in Wnt and Hedgehog signaling. Genes Dev *20*, 399-410.**
**S68. Cadigan, K.M., and Peifer, M. (2009). Wnt signaling from development to disease: insights from model systems. Cold Spring Harbor perspectives in biology *1*, a002881.**
**S69. Siegfried, E., Chou, T.B., and Perrimon, N. (1992). wingless signaling acts through zeste-white 3, the Drosophila homolog of glycogen synthase kinase-3, to regulate engrailed and establish cell fate. Cell *71*, 1167-1179.**
**S70. Bourouis, M., Heitzler, P., el Messal, M., and Simpson, P. (1989). Mutant Drosophila embryos in which all cells adopt a neural fate. Nature *341*, 442-444.**
**S71. McCartney, B.M., McEwen, D.G., Grevengoed, E., Maddox, P., Bejsovec, A., and Peifer, M. (2001). Drosophila APC2 and Armadillo participate in tethering mitotic spindles to cortical actin. Nature cell biology *3*, 933-938.**
**S72. Price, M.A., and Kalderon, D. (2002). Proteolysis of the Hedgehog signaling effector Cubitus interruptus requires phosphorylation by Glycogen Synthase Kinase 3 and Casein Kinase 1. Cell *108*, 823-835.**
**S73. Lee, J.Y. (2009). Versatile casein kinase 1: multiple locations and functions. Plant signaling & behavior *4*, 652-654.**
**S74. Liu, W., Xu, Z.H., Luo, D., and Xue, H.W. (2003). Roles of OsCKI1, a rice casein kinase I, in root development and plant hormone sensitivity. The Plant journal : for cell and molecular biology *36*, 189-202.**
**S75. Ben-Nissan, G., Cui, W., Kim, D.J., Yang, Y., Yoo, B.C., and Lee, J.Y. (2008). Arabidopsis casein kinase 1-like 6 contains a microtubule-binding domain and affects the organization of cortical microtubules. Plant physiology *148*, 1897-1907.**
**S76. Min, L., Zhu, L., Tu, L., Deng, F., Yuan, D., and Zhang, X. (2013). Cotton GhCKI disrupts normal male reproduction by delaying tapetum programmed cell death via inactivating starch synthase. The Plant journal : for cell and molecular biology *75*, 823-835.**
**S77. Kondo, Y., Fujita, T., Sugiyama, M., and Fukuda, H. (2014). A novel system for xylem cell differentiation in Arabidopsis thaliana. Molecular plant.**
**S78. Cai, Z., Liu, J., Wang, H., Yang, C., Chen, Y., Li, Y., Pan, S., Dong, R., Tang, G., Barajas-Lopez Jde, D., et al. (2014). GSK3-like kinases positively modulate abscisic acid signaling through phosphorylating subgroup III SnRK2s in Arabidopsis. Proc Natl Acad Sci U S A *111*, 9651-9656.**
**S79. Qi, X., Chanderbali, A.S., Wong, G.K., Soltis, D.E., and Soltis, P.S. (2013). Phylogeny and evolutionary history of glycogen synthase kinase 3/SHAGGY-like kinase genes in land plants. BMC evolutionary biology *13*, 143.**
**S80. Youn, J.H., Kim, T.W., Kim, E.J., Bu, S., Kim, S.K., Wang, Z.Y., and Kim, T.W. (2013). Structural and functional characterization of Arabidopsis GSK3-like kinase AtSK12. Molecules and cells *36*, 564-570.**
**S81. Zhang, D., Ye, H., Guo, H., Johnson, A., Zhang, M., Lin, H., and Yin, Y. (2014). Transcription factor HAT1 is phosphorylated by BIN2 kinase and mediates brassinosteroid repressed gene expression in Arabidopsis. The Plant journal : for cell and molecular biology *77*, 59-70.**
**S82. Reddi, A.R., and Culotta, V.C. (2013). SOD1 integrates signals from oxygen and glucose to repress respiration. Cell *152*, 224-235.**
**S83. Kafadar, K.A., Zhu, H., Snyder, M., and Cyert, M.S. (2003). Negative regulation of calcineurin signaling by Hrr25p, a yeast homolog of casein kinase I. Genes Dev *17*, 2698-2708.**

**S84.** **Rubin-Bejerano, I., Sagee, S., Friedman, O., Pnueli, L., and Kassir, Y. (2004). The in vivo activity of Ime1, the key transcriptional activator of meiosis-specific genes in Saccharomyces cerevisiae, is inhibited by the cyclic AMP/protein kinase A signal pathway through the glycogen synthase kinase 3-beta homolog Rim11. Mol Cell Biol *24*, 6967-6979.**

**S85.** **Pereira, J., Pimentel, C., Amaral, C., Menezes, R.A., and Rodrigues-Pousada, C. (2009). Yap4 PKA- and GSK3-dependent phosphorylation affects its stability but not its nuclear localization. Yeast *26*, 641-653.**

**S86.** **Ralph, M.R., and Menaker, M. (1988). A mutation of the circadian system in golden hamsters. Science *241*, 1225-1227.**

**S87.** **Lowrey, P.L., Shimomura, K., Antoch, M.P., Yamazaki, S., Zemenides, P.D., Ralph, M.R., Menaker, M., and Takahashi, J.S. (2000). Positional syntenic cloning and functional characterization of the mammalian circadian mutation tau. Science *288*, 483-492.**

**S88.** **Meng, Q.J., Logunova, L., Maywood, E.S., Gallego, M., Lebiecki, J., Brown, T.M., Sladek, M., Semikhodskii, A.S., Glossop, N.R., Piggins, H.D., et al. (2008). Setting clock speed in mammals: the CK1 epsilon tau mutation in mice accelerates circadian pacemakers by selectively destabilizing PERIOD proteins. Neuron *58*, 78-88.**

**S89.** **Xu, Y., Padiath, Q.S., Shapiro, R.E., Jones, C.R., Wu, S.C., Saigoh, N., Saigoh, K., Ptacek, L.J., and Fu, Y.H. (2005). Functional consequences of a CKIdelta mutation causing familial advanced sleep phase syndrome. Nature *434*, 640-644.**

**S90.** **Toh, K.L., Jones, C.R., He, Y., Eide, E.J., Hinz, W.A., Virshup, D.M., Ptacek, L.J., and Fu, Y.H. (2001). An hPer2 phosphorylation site mutation in familial advanced sleep phase syndrome. Science *291*, 1040-1043.**

**S91.** **Lee, H.M., Chen, R., Kim, H., Etchegaray, J.P., Weaver, D.R., and Lee, C. (2011). The period of the circadian oscillator is primarily determined by the balance between casein kinase 1 and protein phosphatase 1. Proc Natl Acad Sci U S A *108*, 16451-16456.**

**S92.** **Meng, Q.J., Maywood, E.S., Bechtold, D.A., Lu, W.Q., Li, J., Gibbs, J.E., Dupre, S.M., Chesham, J.E., Rajamohan, F., Knafels, J., et al. (2010). Entrainment of disrupted circadian behavior through inhibition of casein kinase 1 (CK1) enzymes. Proc Natl Acad Sci U S A *107*, 15240-15245.**

**S93.** **Hirota, T., Lee, J.W., Lewis, W.G., Zhang, E.E., Breton, G., Liu, X., Garcia, M., Peters, E.C., Etchegaray, J.P., Traver, D., et al. (2010). High-throughput chemical screen identifies a novel potent modulator of cellular circadian rhythms and reveals CKIalpha as a clock regulatory kinase. PLoS Biol *8*, e1000559.**

**S94.** **Paul, J.R., Johnson, R.L., Jope, R.S., and Gamble, K.L. (2012). Disruption of circadian rhythmicity and suprachiasmatic action potential frequency in a mouse model with constitutive activation of glycogen synthase kinase 3. Neuroscience *226*, 1-9.**

**S95.** **Iitaka, C., Miyazaki, K., Akaike, T., and Ishida, N. (2005). A role for glycogen synthase kinase-3beta in the mammalian circadian clock. J Biol Chem *280*, 29397-29402.**

**S96.** **Hirota, T., Lewis, W.G., Liu, A.C., Lee, J.W., Schultz, P.G., and Kay, S.A. (2008). A chemical biology approach reveals period shortening of the mammalian circadian clock by specific inhibition of GSK-3beta. Proc Natl Acad Sci U S A *105*, 20746-20751.**

**S97.** **Yin, L., Wang, J., Klein, P.S., and Lazar, M.A. (2006). Nuclear receptor Rev-erbalpha is a critical lithium-sensitive component of the circadian clock. Science *311*, 1002-1005.**

**S98.** **Sahar, S., Zocchi, L., Kinoshita, C., Borrelli, E., and Sassone-Corsi, P. (2010). Regulation of BMAL1 protein stability and circadian function by GSK3beta-mediated phosphorylation. PLoS One *5*, e8561.**

**S99.** **Spengler, M.L., Kuropatwinski, K.K., Schumer, M., and Antoch, M.P. (2009). A serine cluster mediates BMAL1-dependent CLOCK phosphorylation and degradation. Cell Cycle *8*, 4138-4146.**

**S100.** **Price, J.L., Blau, J., Rothenfluh, A., Abodeely, M., Kloss, B., and Young, M.W. (1998). double-time is a novel Drosophila clock gene that regulates PERIOD protein accumulation. Cell *94*, 83-95.**

**S101.** **Kloss, B., Price, J.L., Saez, L., Blau, J., Rothenfluh, A., Wesley, C.S., and Young, M.W. (1998). The Drosophila clock gene double-time encodes a protein closely related to human casein kinase Iepsilon. Cell *94*, 97-107.**

**S102.** **Martinek, S., Inonog, S., Manoukian, A.S., and Young, M.W. (2001). A role for the segment polarity gene shaggy/GSK-3 in the Drosophila circadian clock. Cell *105*, 769-779.**

**S103.** **Harms, E., Young, M.W., and Saez, L. (2003). CK1 and GSK3 in the Drosophila and mammalian circadian clock. Novartis Foundation symposium *253*, 267-277; discussion 102-269, 277-284.**

**S104.** **O'Neill, J.S., van Ooijen, G., Dixon, L.E., Troein, C., Corellou, F., Bouget, F.Y., Reddy, A.B., and Millar, A.J. (2011). Circadian rhythms persist without transcription in a eukaryote. Nature *469*, 554-558.**

**S105.** **van Ooijen, G., Hindle, M., Martin, S.F., Barrios-Llerena, M., Sanchez, F., Bouget, F.Y., O'Neill, J.S., Le Bihan, T., and Millar, A.J. (2013). Functional analysis of Casein Kinase 1 in a minimal circadian system. PLoS One *8*, e70021.**

**S106.** **Tan, S.T., Dai, C., Liu, H.T., and Xue, H.W. (2013). Arabidopsis casein kinase1 proteins CK1.3 and CK1.4 phosphorylate cryptochrome2 to regulate blue light signaling. The Plant cell *25*, 2618-2632.**

**S107.** **Gorl, M., Merrow, M., Huttner, B., Johnson, J., Roenneberg, T., and Brunner, M. (2001). A PEST-like element in FREQUENCY determines the length of the circadian period in Neurospora crassa. The EMBO journal *20*, 7074-7084.**

**S108.** **Huang, G., Chen, S., Li, S., Cha, J., Long, C., Li, L., He, Q., and Liu, Y. (2007). Protein kinase A and casein kinases mediate sequential phosphorylation events in the circadian negative feedback loop. Genes Dev *21*, 3283-3295.**

**S109.** **He, Q., Cha, J., He, Q., Lee, H.C., Yang, Y., and Liu, Y. (2006). CKI and CKII mediate the FREQUENCY-dependent phosphorylation of the WHITE COLLAR complex to close the Neurospora circadian negative feedback loop. Genes Dev *20*, 2552-2565.**

**S110.** **Tataroglu, O., Lauinger, L., Sancar, G., Jakob, K., Brunner, M., and Diernfellner, A.C. (2012). Glycogen synthase kinase is a regulator of the circadian clock of Neurospora crassa. J Biol Chem *287*, 36936-36943.**

**S111.** **van Dijken, J.P., Bauer, J., Brambilla, L., Duboc, P., Francois, J.M., Gancedo, C., Giuseppin, M.L., Heijnen, J.J., Hoare, M., Lange, H.C., et al. (2000). An interlaboratory comparison of physiological and genetic properties of four Saccharomyces cerevisiae strains. Enzyme and microbial technology *26*, 706-714.**
