# Supplementary material for: Metabolic Cycles in Yeast Share Features Conserved among Circadian Rhythms
Source: Curr Biol. 2015 Apr 20;25(8):1056–62. doi: 10.1016/j.cub.2015.02.035 (PMC4406945; doi:10.1016/j.cub.2015.02.035)
Supplement: Document S2. Article plus Supplemental Information [file mmc2.pdf]

Report

Current Biology

Metabolic Cycles in Yeast Share Features Conserved among Circadian Rhythms

Highlights

- Yeast respiratory oscillations (YROs) share features with circadian rhythms
- Changes that alter the period of circadian rhythms have the same effect on YROs
- Oxidation cycles of peroxiredoxins are a characteristic of both oscillations
- Mechanistic similarities between these cycles may reflect a common origin

Authors

Helen C. Causton, Kevin A. Feeney, Christine A. Ziegler, John S. O'Neill

Correspondence

hc2415@columbia.edu (H.C.C.),
oneillj@mrc-lmb.cam.ac.uk (J.S.O.)

In Brief

The clock gene feedback loops thought to drive circadian rhythms are not conserved across eukaryotes, but, perplexingly, several enzymes that determine clock speed are. Causton et al. now show that respiratory cycles in yeast share some key features with the clock in mammalian cells, raising questions about the origins of biological timekeeping.

Causton et al., 2015, Current Biology *25*, 1056–1062
April 20, 2015 ©2015 The Authors
http://dx.doi.org/10.1016/j.cub.2015.02.035

CellPress

Current Biology
Report

Metabolic Cycles in Yeast Share Features Conserved among Circadian Rhythms

Helen C. Causton,[1,*] Kevin A. Feeney,[2] Christine A. Ziegler,[1,3] and John S. O'Neill[2,*]
[1]Department of Biological Sciences, Columbia University, 617 Fairchild Building, 1212 Amsterdam Avenue, Mail Code 2442, New York, NY 10027, USA
[2]MRC Laboratory of Molecular Biology, Francis Crick Avenue, Cambridge CB2 0QH, UK
[3]Present address: Department of Chemistry, University of Rochester, Rochester, NY 14627, USA
*Correspondence: hc2415@columbia.edu (H.C.C.), oneillj@mrc-lmb.cam.ac.uk (J.S.O.)
http://dx.doi.org/10.1016/j.cub.2015.02.035
This is an open access article under the CC BY license (http://creativecommons.org/licenses/by/4.0/).

SUMMARY

Cell-autonomous circadian rhythms allow organisms to temporally orchestrate their internal state to anticipate and/or resonate with the external environment [1, 2]. Although ∼24-hr periodicity is observed across aerobic eukaryotes, the central mechanism has been hard to dissect because few simple models exist, and known clock proteins are not conserved across phylogenetic kingdoms [1, 3, 4]. In contrast, contributions to circadian rhythmicity made by a handful of post-translational mechanisms, such as phosphorylation of clock proteins by casein kinase 1 (CK1) and glycogen synthase kinase 3 (GSK3), appear conserved among phyla [3, 5]. These kinases have many other essential cellular functions and are better conserved in their contribution to timekeeping than any of the clock proteins they phosphorylate [6]. Rhythmic oscillations in cellular redox state are another universal feature of circadian timekeeping, e.g., over-oxidation cycles of abundant peroxiredoxin proteins [7–9]. Here, we use comparative chronobiology to distinguish fundamental clock mechanisms from species and/or tissue-specific adaptations and thereby identify features shared between circadian rhythms in mammalian cells and non-circadian temperature-compensated respiratory oscillations in budding yeast [10]. We find that both types of oscillations are coupled with the cell division cycle, exhibit period determination by CK1 and GSK3, and have peroxiredoxin over-oxidation cycles. We also explore how peroxiredoxins contribute to YROs. Our data point to common mechanisms underlying both YROs and circadian rhythms and suggest two interpretations: either certain biochemical systems are simply permissive for cellular oscillations (with frequencies from hours to days) or this commonality arose via divergence from an ancestral cellular clock.

RESULTS AND DISCUSSION

A Conserved Cell-Cycle Regulator, Swe1, Also Regulates the YRO

In order to understand why certain ubiquitous post-translational mechanisms have a highly conserved circadian clock function, we investigated their contribution to the shorter period (∼1–5 hr, ultradian) yeast respiratory oscillations (YROs) in *Saccharomyces cerevisae*, which lack robust circadian rhythms and canonical clock proteins [11]. The YRO is a cell-autonomous, temperature-compensated rhythm in oxygen consumption that synchronizes spontaneously when cells are grown at high density in aerobic, nutrient-limited, continuous culture [10, 12–14] (Figure 1A). Beyond the difference in oscillation frequency (∼1 day^{-1} versus ∼8 day^{-1}), circadian rhythms and YROs are highly similar (summarized in Table S1). In animal cells, the circadian clock regulates the timing of cell division [15], and in rapidly proliferating cells, the cell division cycle (CDC) and circadian cycle can become tightly coupled [16]. This is achieved, in part, through daily rhythms in the expression of a conserved cell-cycle regulator, the Ser/Thr kinase Wee1 [17, 18]. The homolog of Wee1 in budding yeast, Swe1, functions at the G2/M checkpoint, with additional roles during G_1 [19]. Like the circadian cycle, the YRO does not require cell division [20] but does gate DNA replication so that S-phase entry does not typically occur during the oxidative portion of the YRO (OX) [21]. We asked whether coupling between the YRO and the CDC in yeast might occur via Swe1 in the same way that Wee1 connects circadian rhythms and the CDC in mammalian cells.

SWE1 was deleted and synchronized respiratory oscillations were initiated in a bioreactor. The *swe1* strain underwent YROs with significantly shorter period than wild-type (Figures 1B–1D) but grew more slowly (44% ± 10% of wild-type; Figure S1D), confirming that the relationship between YRO and CDC is not fixed [20] and is regulated by Swe1. The proportion of $G_{1/0}$ cells relative to cells with replicated DNA is represented by the 1C:2C ratio and peaks at the end of OX [12, 21]. The amplitude of 1C:2C across the oscillation was significantly attenuated in the mutant, with approximately half as many cells leaving G_1 each oscillation compared with wild-type (Figures 1C, 1D, S1A, and S1B) and a higher percentage of cells in S/G_2/M overall (Figure S1C). The YRO is specific to $G_{1/0}$ [20], so it is likely that the faster respiratory oscillation of the *swe1* strain results from the relatively shorter

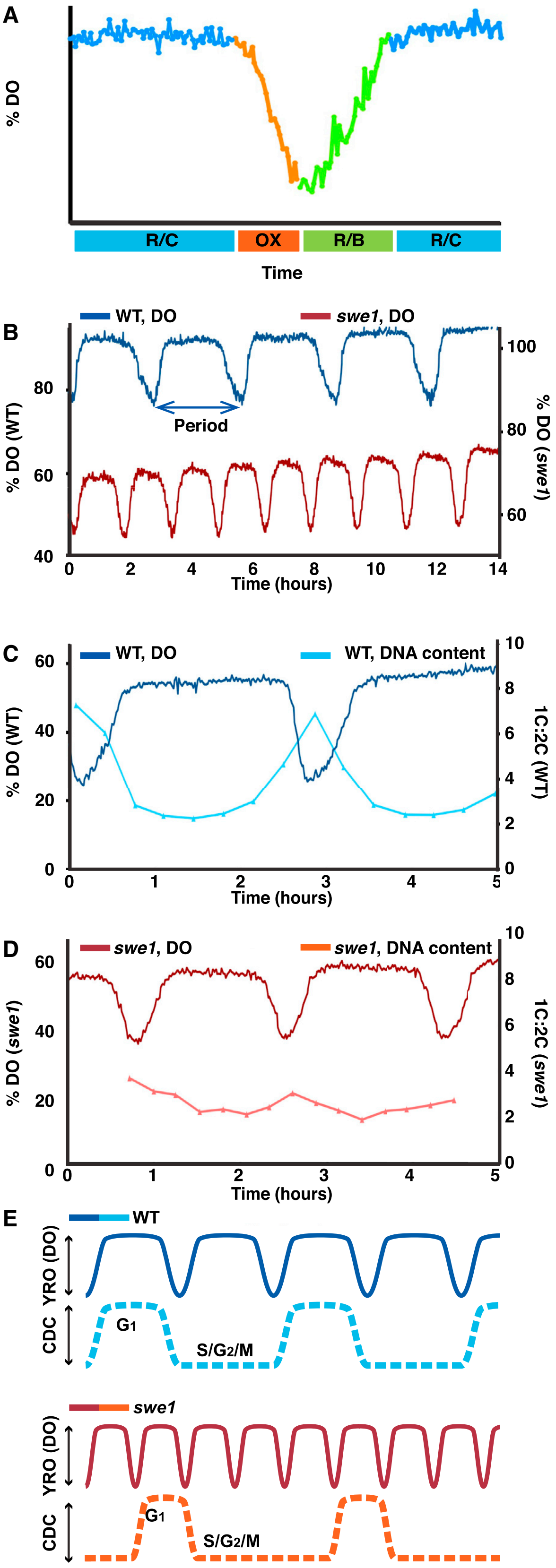

Figure 1. The CDC and YROs Are Affected by Deletion of *SWE1*

(A) Phases of the oscillation referred to throughout the text: reductive/charging (R/C), respiratory or oxidative (OX), and reductive building (RB).

(B) Dissolved oxygen (DO) trace showing that wild-type strains cycle with YRO (period 2.77 hr, SD 0.26, n = 17), whereas *swe1* strains cycle substantially faster (t test, $p < 0.001$; period 1.62 hr, SD 0.11, n = 12). See also Figure S1.

(C and D) Dissolved oxygen traces are highly synchronized with DNA content in wild-type strains and *swe1* strains.

(E) Model inferred from population-based data illustrating the relationship between the YRO and the CDC for wild-type and *swe1* strains. The dotted line represents the CDC of a single cell within the population. The population is not synchronized with respect to cell division, but the YRO gates when a cell can enter S phase.

duration of $G_{1/0}$ in the mutant. Our data thus support a model in which the YRO and the CDC remain coupled in the *swe1* mutant, but the cell cycle is longer and fewer cells are undergoing respiratory oscillations at any point in time (Figure 1E). As mammalian cancer cell lines exhibit a wider range of circadian periods when assessed in vitro than do primary cells, we wonder whether altered circadian timekeeping might constitute a more general hallmark of cells that have lost tumor suppressor genes such as *WEE1*.

CK1 and GSK3 Determine Period Length in Both the YRO and Mammalian Cellular Clock

CK1 and GSK3 are two families of ubiquitous eukaryotic Serine/Threonine kinases that regulate a broad range of cellular processes, including metabolism, cell migration, and *wnt* signaling (Table S2). These enzymes act to regulate target protein stability, subcellular localization, and complex formation and can act synergistically with each other, e.g., in the regulation of β-catenin. Both kinases also play a conserved role in setting the speed of the circadian clock, although their targets are not conserved (Table S2) [22, 23]. We postulated that these enzymes similarly contribute to the speed of the YRO. We tested this by pharmacological inhibition of yeast CK1δ/ε homologs using the selective inhibitors PF670462 and LH846 [23, 24] and by knocking out yeast GSK3β homolog, *RIM11*. In parallel, we performed experiments with mouse fibroblasts expressing transcriptional or translational clock gene::luciferase (*per2:luc* or PER2::LUC) reporters (Figures 2 and S2). In both cases, our hypothesis was confirmed: CK1 inhibition dose dependently increased the period of the YRO and the mammalian clock, whereas GSK3β knockout significantly shortened the period of both. These results show that perturbation of the yeast CK1 and GSK homologs has similar effects to those observed for circadian rhythms in diverse species. Although we do not yet know which kinase substrates are relevant, our data are consistent with a model in which they play similar roles.

Oscillations in the Redox State of Peroxiredoxin Tsa1 Accompanies the YRO

Peroxiredoxins (PRXs) are abundant thiol-specific cellular peroxidases that employ a conserved cysteine residue for the reduction of intracellular peroxides. Oxidized PRX usually dimerizes via a disulphide and is re-reduced by the thioredoxin system or may become over-oxidized to the sulphinic form (SO_2) and subsequently recycled by sulphiredoxin (Srx) [25]. These ubiquitous antioxidants were recently suggested to constitute universal

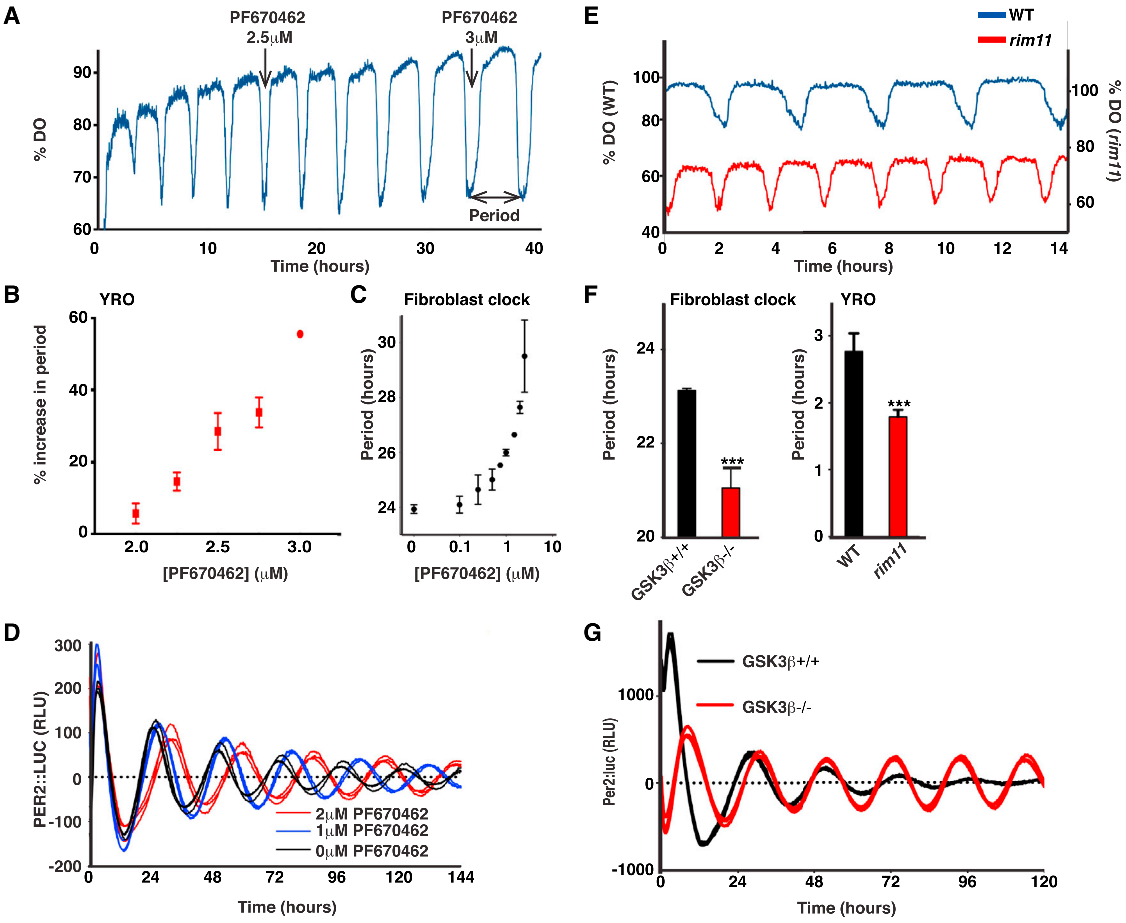

Figure 2. Perturbations that Affect the Period of Circadian Rhythms Have a Similar Effect on YROs

(A) Representative DO traces showing that pharmacological inhibition of casein kinase I in yeast increases the period of oscillation.

(B) Grouped data showing the dose dependence of CK1 inhibition on the YRO (mean ± SEM, one-way ANOVA, $p = 0.0164$). Although the target of PF670462 has not been characterized in yeast, addition of LH846, another CK1 inhibitor, had a similar effect (Figure S2). These results suggest that the period increase is due to direct inhibition of yeast CK1.

(C) Grouped data showing dose-dependent period lengthening on circadian period in immortalized mouse fibroblasts (mean ± SD, $n = 4$, $p < 0.0001$ for concentration effect by two-way ANOVA).

(D) PF670462 has a dose-dependent effect on circadian period in mouse fibroblasts as reported previously. Representative detrended bioluminescence traces are shown.

(E) Representative data DO traces showing that a yeast strain deleted for *RIM11*, homolog of GSK3, has a shorter period of oscillation.

(F) Bar graph showing the effect (mean ± SEM) of homozygous deletion of GSK3β on the period of the circadian cellular oscillation in fibroblasts ($n = 4$) and deletion of *RIM11* on the YRO ($n = 17$); $p < 0.001$ by unpaired t test in both cases.

(G) Homozygous deletion of GSK3β shortens circadian period in mouse embryonic fibroblasts. Representative detrended traces are shown.

Asterisks represent different p value thresholds (throughout): *$p < 0.05$; **$p < 0.01$; ***$p < 0.001$.

markers for circadian rhythms, as they exhibit a ~24-hr rhythm in cysteine over-oxidation that persists (albeit perturbed) in circadian clock mutants and also in the absence of nascent gene expression, e.g., in mammalian erythrocytes [8, 26]. Although PRX over-oxidation cycles are thought to reflect an underlying oscillation in cytosolic redox balance, it is unclear whether PRX activity is required for clock function. The YRO coordinates with many cellular processes (Table S1), including mitochondrial and cytosolic redox metabolism. We therefore postulated that if PRX over-oxidation reflects a rhythm in the production of reactive oxygen species (ROS) and/or reducing equivalents, PRX oxidation should also be driven by the YRO. *S. cerevisiae* expresses three PRXs that contain the conserved 9-mer motif recognized by commercial antisera: Tsa1, Tsa2, and mitochondrial Prx1 (Figure 3A). By using single gene deletions and peroxide treatment, we observed oxidation-specific bands at the expected molecular weight (~22 kD) only in strains with wild-type *TSA1*, indicating that the anti-PRX-$SO_{2/3}$ antiserum specifically recognizes over-oxidized Tsa1 (Figure 3B). Samples were then collected over the course of the YRO, run at two different dilution rates in three independent experiments, to test whether PRX over-oxidation correlated with YROs of different period, and analyzed by western blotting. The PRX-$SO_{2/3}$ signal showed a fixed-phase relationship with the YRO,

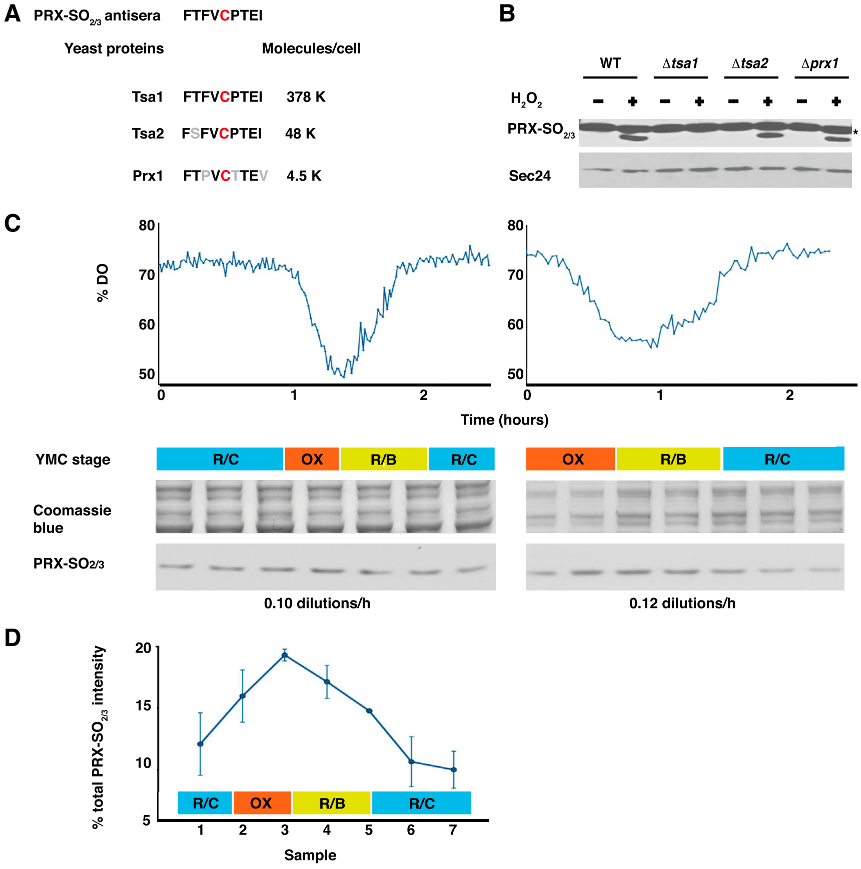

Figure 3. PRX Tsa1 Undergoes Cycles of Oxidation across the YRO

(A) Three PRXs in the yeast *S. cerevisiae* have the nine amino acid sequence recognized by the PRX-$SO_{2/3}$ antisera when over-oxidized.

(B) Western blot showing that over-oxidized Tsa1 is recognized by the PRX-$SO_{2/3}$ antisera in a redox-dependent manner. Samples were harvested immediately before (–) or 15 min after (+) the addition of hydrogen peroxide (1 mM final concentration). The asterisk marks a cross-reacting band.

(C) YROs were obtained using 0.1 or 0.12 dilutions/hr (sampled every 20 or 12 min, respectively) and monitored using the dissolved oxygen trace (top). Bottom: whole-cell extracts obtained from samples taken across the oscillation were analyzed for PRX over-oxidation by western blotting.

(D) Grouped data showing the normalized PRX-$SO_{2/3}$ intensity over the YRO, peaking around late OX phase (mean ± SEM, n = 3, p = 0.024 for time effect by two-way ANOVA).

consistently peaking just after the end of oxidative phase (OX, Figure 1D), when ROS generated as respiratory by-products are likely to be maximal (Figures 3C and 3D).

PRX Activity Affects, but Is Not Required for, Ultradian or Circadian Rhythms

To test whether PRX activity is necessary for the YRO, we knocked out the major cytosolic PRXs, *TSA1* and *TSA2*, and also *PRX1*. Since *TSA1* and *TSA2* act cooperatively and/or semi-redundantly, we also tested a *tsa1 tsa2* strain and an *srx1* strain in order to establish whether catalytic cycling of PRX over-oxidation might be important. All of the strains tested underwent respiratory oscillations, showing that PRX activity is not required for cycling (Figures 4 and S3B–S3F). However, Tsa1 and Tsa2 together make some contribution to the integrity of this temporal metabolic program because the double deletion strain has a distinct dissolved oxygen profile, with a pronounced "dip" in the trace during the reductive phase and cycles with a slightly shorter period (Figure 4A). This dip may reflect a transient respiratory burst during the "reductive/charging" (R/C) portion of the cycle, suggesting that one function of Tsa1/2 may be to stably maintain reductive metabolism. Catalytic recycling of Tsa1/2 by Srx1 makes no YRO contribution, as the *srx1* strain is phenotypically similar to wild-type (Figures S3E and S3F). Consistent with this result, circadian PRX over-oxidation cycles persist in red blood cells lacking Srx [27] but require proteasomal activity [27], and we speculate PRX-$SO_{2/3}$ may similarly be degraded by the 20S proteasome. We also note that a strain lacking Prx1 exhibits a lengthened period (Figure S3F), suggesting that antioxidant mitochondrial balance also contributes to this respiratory oscillation, as might be expected for an oscillation with a redox cycle at its core [13].

The equivalent PRX loss-of-function experiment in mammalian cells is impractical as there are six PRX isoforms, so we used conoidin A (CA), a naturally occurring, membrane-permeable irreversible (2-cys) PRX inhibitor that reacts with the catalytic (peroxidatic) cysteine residue [28]. We confirmed CA activity by observing that it dose dependently blocks over-oxidation of cellular PRX in mouse fibroblasts following 30-min treatment with 2 mM H_2O_2 (Figure S3). Circadian bioluminescence assays revealed that at sub-toxic concentrations (≤5 μM), PRX inactivation subtly, but significantly, shortened the period of oscillation, accompanied by much larger dose-dependent effects on the amplitude and phase of PERIOD2::LUCIFERASE rhythms (Figures 4B–4E). This result echoes previous observations of circadian activity in 2-cys PRX null mutants in *Synechococcus elongatus* (a cyanobacterium) and *Arabidopsis thaliana* (a plant) [8]. At higher CA concentrations (>5 μM), cells were dead within 24 hr. These results suggest that PRX activity modulates, but is not required for, cellular circadian rhythmicity. These results are similar to those observed for YROs.

Comparative Chronobiology Offers New Insights toward Mechanism

Collectively, our data show that conserved post-translational features of circadian rhythms in eukaryotes are also a feature of YROs in an organism not known for 24-hr periodicity and that YROs are not merely a function of the CDC. In our view, these results are consistent with two different models. (1) There are a number of conserved cellular mechanisms involved in

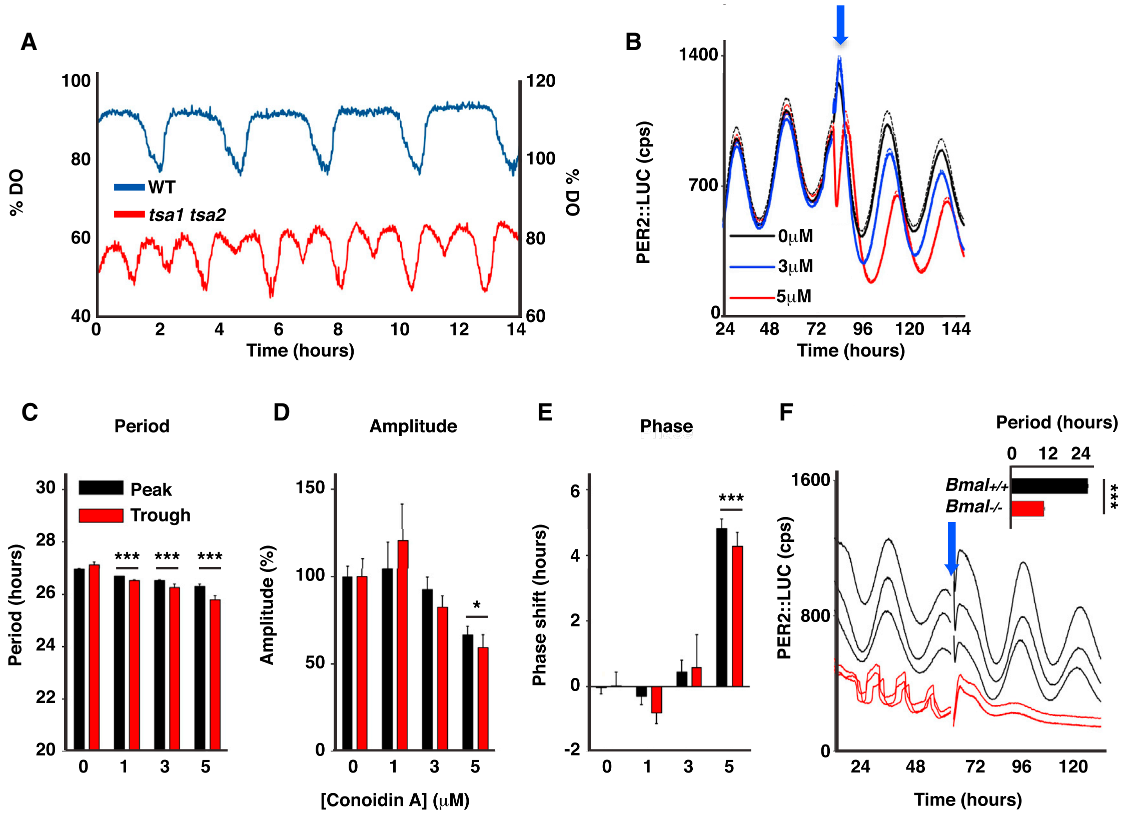

Figure 4. PRXs Contribute to YROs but Are Not Required for Cycling

(A) Dissolved oxygen trace for a wild-type strain (period 2.77 hr, SD 0.26, n = 17) and a *tsa1 tsa2* double mutant strain (period 2.37 hr, SD 0.18, n = 6, $p < 0.001$).

(B) Grouped detrended bioluminescence traces (mean ± SEM, n = 3) showing the effect of a 2-cys PRX inhibitior on PER2::LUCIFERASE rhythms in immortalized mouse fibroblasts added at the peak or trough (data not shown). The blue arrow indicates the point at which conoidin A or vehicle was added.

(C–E) Conoidin A subtly shortens circadian period ($p < 0.0001$; C) and has robust effects upon amplitude (p = 0.0024; D) and circadian phase ($p < 0.0001$; E). Mean ± SEM is shown for (C)–(E) (n = 3, p values are two-way ANOVA, concentration effect). Asterisks report Bonferroni post-test p values for each drug concentration versus vehicle control.

(F) Immortalized *Bmal1*$^{-/-}$ fibroblasts exhibit an ultradian rhythm of PER2::LUCIFERASE activity under certain culture conditions, abolished following a complete media change. Inset shows mean period ± SEM before the media change (unpaired t test versus wild-type controls, $p < 0.0001$, n = 16).

numerous cellular functions that are also permissive for biological oscillations in the frequency range of hours. These enzymes do not have a specific role in the circadian clock but have a more general cellular function. (2) Yeast respiratory oscillations rely upon the same central timekeeping mechanisms employed by circadian clocks in higher organisms, and this reflects a common origin.

In the first model, recruitment of the same post-translational mechanisms to sustain biological rhythms of quite different periods means that they are the chronobiological equivalent of housekeeping enzymes. This could explain why, for example, CK1 activity also determines the period of circatidal rhythms in *Eurydice pulchra*, which is driven by a non-circadian clock [29]. The second model would be supported if *S. cerevisiae* evolved under conditions where selection favored rapid growth over circadian timing. Budding yeast is known to have undergone genome duplications followed by massive gene loss that would have facilitated this process [30]. The benefits conferred by clock-controlled temporal segregation of metabolism and gene expression would continue to bestow a fitness advantage, with the consequence that the period of the oscillation shortened to allow a faster cell cycle. In support of the possibility that a circadian clock became the YRO through gene deletion, we were interested to observe that under certain conditions, fibroblasts homozygous null for the "core clock gene" Bmal1 exhibit a pronounced ultradian rhythm in "clock gene" expression (Figure 4F), echoing previous observations made using pacemaker neurons from Bmal1 null mice [31].

In either case, by comparing similar biological rhythms with different periods in very distantly related eukaryotes (yeast versus mouse), our approach offers the potential to identify the processes that determine the speed at which biological clocks run. The Last Eukaryotic Common Ancestor (LECA) possessed mitochondria, a nucleus, metabolic pathways that included glycolysis, and the pentose phosphate pathway, as well as a cell cycle regulated by cyclins and cyclin-dependent kinases [32, 33]. We speculate that it also had a circadian clock.

EXPERIMENTAL PROCEDURES

Strain Construction and Growth Curves in Yeast

Yeast deletion strains were made by insertion of the KanMX and/or NatMX cassette in the CEN.PK113-7D background [34] using standard genetic methods (Table S3).

The optical density (OD) of wild-type and *swe1* strains was measured over time, and a corrected value was obtained by subtracting background density (OD of media at each time point). Relative growth rates were determined by linear regression during log phase growth in batch cultures. Cell number was counted using a haemocytometer, and turbidity measurements were obtained using a Bioscreen C machine (Lab Systems). These experiments were carried out at 30°C using the same media as that used to feed the bioreactor.

Protein Preparation and Detection

Yeast whole-cell extracts were prepared by trichloroacetic acid (TCA) precipitation as described [35], with the addition of the following protease inhibitors (Sigma): aminobenzamide dihydrochloride (200 μg/ml), antipain (1 μg/ml), aprotinin (1 μg/ml), leupeptin (1 μg/ml), chymostatin (1 μg/ml), PMSF (200 μg/ml), TPCK (50 μg/ml), and pepstatin (1 μg/ml). Gel electrophoresis and western blotting were carried out as described [8], except that mini NuPAGE gels (Life Technologies) were used and proteins were wet transferred to PVDF. PRX-$SO_{2/3}$ antibody (ab16830) was purchased from Abcam.

YROs

Respiratory oscillations were generated as described [10, 36], using a 7.5 L New Brunswick Celligen 115/Bioreactor containing 2 L media at pH 3.4 at 30°C with 4 L/min aeration, 550 rpm agitation. The pH was maintained with 10% NaOH. Unless otherwise stated, all experiments used a continuous flow rate of 0.1 dilutions/hr. The oxygen probe was calibrated prior to each experiment. All experiments on mutant strains were conducted at least twice, while those involving dose response curves represent the combined results from two (LH846) or three (PF670462) experiments. PF670462 and LH846 were purchased from Tocris Bioscience.

Flow Cytometry

DNA was stained using propidium iodide (PI) using standard methods. Flow cytometry was carried out using a FACSCalibur flow cytometer (Becton Dickinson). 20,000 cells were scored for each sample.

Data Analysis

Period length was calculated using a custom-built script in MATLAB. To automatically identify minima, we used a sliding window smoothing algorithm on the dissolved oxygen data to reduce measurement noise. All local minima points were then identified in the smoothed data. A period was defined between each pair of adjacent minima points. t tests were carried out in Excel, using the T.TEST function for two-tailed samples of unequal variance. NIH ImageJ software was used to quantify the intensity of bands on western blots and protein gels. Other statistical analyses were performed using Graphpad Prism. Intensities from western blotting were corrected for variations in total protein concentration in each lane and standardized based on total signal intensity across the oscillation.

Culture and Manipulation of Mammalian Cells

Primary fibroblasts homozygous for PERIOD2::LUCIFERASE [37] were isolated from the lung tissue of adult males and cultured as described previously [38] and then immortalized by serial passage [39]. GSK3$\beta^{-/-}$ and wild-type control mouse embryonic fibroblasts from [40] were stably transfected with a plasmid encoding *Per2:luc*. Passage number did not exceed 20. All animal work was licensed under the UK Animals (Scientific Procedures) Act of 1986 with local ethical approval. Cell lysis and immunoblotting is described in the legend for Figure S3.

Monitoring of Circadian Rhythms

Cells were seeded at a density of 10^5 per 35-mm dish and grown to complete confluence with regular media changes as described previously [38]. Bioluminescence assays were performed in HEPES-buffered "Air Medium" [38] supplemented with 10% HyClone FetalClone III serum, 1 mM luciferin, 2% B-27 supplement, and 1× Glutamax in all cases, with the exception that 1% serum was used in the recording from Bmal1$^{-/-}$ fibroblasts and wild-type controls, and these cells were cultured under 12 hr:12 hr 32°C:37°C temperature cycles for 2 weeks prior to changing to Air Medium for the recording at constant 37°C. Drugs were purchased from Cayman Chemical, dissolved in DMSO, and then diluted into Air Medium such that the final concentration of DMSO did not exceed 0.1%. Within each experiment, DMSO concentration was internally controlled (i.e., equal DMSO at 0 μM and 1 μM drug). Mammalian bioluminescence experiments were performed using a Lumicycle (Actimetrics) at constant 37°C. Lumicycle data were detrended to remove baseline changes and then fit with a damped sine wave in order to determine circadian period, amplitude and phase as in [22]. This and all other statistical analyses were performed using Graphpad Prism.

SUPPLEMENTAL INFORMATION

Supplemental Information includes three figures and three tables and can be found with this article online at http://dx.doi.org/10.1016/j.cub.2015.02.035.

AUTHOR CONTRIBUTIONS

H.C.C. and J.S.O. designed the study and wrote the paper. H.C.C. and C.A.Z. generated data in yeast. J.S.O. and K.A.F. performed experiments in mammalian cells.

ACKNOWLEDGMENTS

We thank Liz Miller and members of her laboratory for useful discussions, critical reading of the manuscript, laboratory resource, and the anti-Sec24 antisera; Jian Yang and Minghui Li for assistance and use of the bioreactor; and Oren Litvin for the MATLAB script used for period determination. At the MRC LMB, we are grateful to the Biomedical Services Group for animal care; Mick Hastings and Jo Chesham for supplying mouse tissue; Jo Westmoreland of MRC-LMB Visual Aids for assistance with figures; Ned Hoyle, Priya Crosby, and Sean Munro for useful discussion; Marrit Putker for critical reading of the manuscript; and Akhilesh Reddy (University of Cambridge). GSK3$\beta^{-/-}$ and wild-type control mouse embryonic fibroblasts were kind gifts from Jim Woodgett (Lunenfeld-Tanenbaum Research Institute, University of Toronto). The CEN.PK113-7D strain was a kind gift from Peter Kötter (Johann Wolfgang Goethe University, Frankfurt am Main). Finally, we are very grateful for the pivotal contribution made by our peer reviewers, whose suggestions and comments were most constructive and insightful. C.A.Z. was supported by the Amgen Scholars Program. J.S.O. is supported by the Medical Research Council (MC_UP_1201/4) and the Wellcome Trust (093734/Z/10/Z).

Received: September 12, 2014
Revised: December 17, 2014
Accepted: February 9, 2015
Published: April 9, 2015

REFERENCES

1. Dunlap, J.C. (1999). Molecular bases for circadian clocks. Cell *96*, 271–290.
2. Roenneberg, T., and Merrow, M. (2005). Circadian clocks - the fall and rise of physiology. Nat. Rev. Mol. Cell Biol. *6*, 965–971.
3. Hastings, M.H., Maywood, E.S., and O'Neill, J.S. (2008). Cellular circadian pacemaking and the role of cytosolic rhythms. Curr. Biol. *18*, R805–R815.
4. Lakin-Thomas, P.L. (2006). Transcriptional feedback oscillators: maybe, maybe not.... J. Biol. Rhythms *21*, 83–92.
5. O'Neill, J.S., van Ooijen, G., Dixon, L.E., Troein, C., Corellou, F., Bouget, F.Y., Reddy, A.B., and Millar, A.J. (2011). Circadian rhythms persist without transcription in a eukaryote. Nature *469*, 554–558.
6. O'Neill, J.S., Maywood, E.S., and Hastings, M.H. (2013). Cellular mechanisms of circadian pacemaking: beyond transcriptional loops. Handbook Exp. Pharmacol. *217*, 67–103.

7. Brody, S., and Harris, S. (1973). Circadian rhythms in neurospora: spatial differences in pyridine nucleotide levels. Science *180*, 498–500.
8. Edgar, R.S., Green, E.W., Zhao, Y., van Ooijen, G., Olmedo, M., Qin, X., Xu, Y., Pan, M., Valekunja, U.K., Feeney, K.A., *et al*. (2012). Peroxiredoxins are conserved markers of circadian rhythms. Nature *485*, 459–464.
9. Bass, J. (2012). Circadian topology of metabolism. Nature *491*, 348–356.
10. Tu, B.P., Kudlicki, A., Rowicka, M., and McKnight, S.L. (2005). Logic of the yeast metabolic cycle: temporal compartmentalization of cellular processes. Science *310*, 1152–1158.
11. Eelderink-Chen, Z., Mazzotta, G., Sturre, M., Bosman, J., Roenneberg, T., and Merrow, M. (2010). A circadian clock in Saccharomyces cerevisiae. Proc. Natl. Acad. Sci. USA *107*, 2043–2047.
12. Klevecz, R.R., Bolen, J., Forrest, G., and Murray, D.B. (2004). A genome-wide oscillation in transcription gates DNA replication and cell cycle. Proc. Natl. Acad. Sci. USA *101*, 1200–1205.
13. Lloyd, D. (2008). Respiratory oscillations in yeasts. Adv. Exp. Med. Biol. *641*, 118–140.
14. Silverman, S.J., Petti, A.A., Slavov, N., Parsons, L., Briehof, R., Thiberge, S.Y., Zenklusen, D., Gandhi, S.J., Larson, D.R., Singer, R.H., and Botstein, D. (2010). Metabolic cycling in single yeast cells from unsynchronized steady-state populations limited on glucose or phosphate. Proc. Natl. Acad. Sci. USA *107*, 6946–6951.
15. Nagoshi, E., Saini, C., Bauer, C., Laroche, T., Naef, F., and Schibler, U. (2004). Circadian gene expression in individual fibroblasts: cell-autonomous and self-sustained oscillators pass time to daughter cells. Cell *119*, 693–705.
16. Kowalska, E., Ripperger, J.A., Hoegger, D.C., Bruegger, P., Buch, T., Birchler, T., Mueller, A., Albrecht, U., Contaldo, C., and Brown, S.A. (2013). NONO couples the circadian clock to the cell cycle. Proc. Natl. Acad. Sci. USA *110*, 1592–1599.
17. Matsuo, T., Yamaguchi, S., Mitsui, S., Emi, A., Shimoda, F., and Okamura, H. (2003). Control mechanism of the circadian clock for timing of cell division in vivo. Science *302*, 255–259.
18. Reddy, A.B., Wong, G.K., O'Neill, J., Maywood, E.S., and Hastings, M.H. (2005). Circadian clocks: neural and peripheral pacemakers that impact upon the cell division cycle. Mutat. Res. *574*, 76–91.
19. Saracino, F., Bassler, J., Muzzini, D., Hurt, E., and Agostoni Carbone, M.L. (2004). The yeast kinase Swe1 is required for proper entry into cell cycle after arrest due to ribosome biogenesis and protein synthesis defects. Cell Cycle *3*, 648–654.
20. Slavov, N., Macinskas, J., Caudy, A., and Botstein, D. (2011). Metabolic cycling without cell division cycling in respiring yeast. Proc. Natl. Acad. Sci. USA *108*, 19090–19095.
21. Chen, Z., Odstrcil, E.A., Tu, B.P., and McKnight, S.L. (2007). Restriction of DNA replication to the reductive phase of the metabolic cycle protects genome integrity. Science *316*, 1916–1919.
22. Hirota, T., Lewis, W.G., Liu, A.C., Lee, J.W., Schultz, P.G., and Kay, S.A. (2008). A chemical biology approach reveals period shortening of the mammalian circadian clock by specific inhibition of GSK-3beta. Proc. Natl. Acad. Sci. USA *105*, 20746–20751.
23. Meng, Q.J., Maywood, E.S., Bechtold, D.A., Lu, W.Q., Li, J., Gibbs, J.E., Dupré, S.M., Chesham, J.E., Rajamohan, F., Knafels, J., *et al*. (2010). Entrainment of disrupted circadian behavior through inhibition of casein kinase 1 (CK1) enzymes. Proc. Natl. Acad. Sci. USA *107*, 15240–15245.
24. Lee, J.W., Hirota, T., Peters, E.C., Garcia, M., Gonzalez, R., Cho, C.Y., Wu, X., Schultz, P.G., and Kay, S.A. (2011). A small molecule modulates circadian rhythms through phosphorylation of the period protein. Angew. Chem. Int. Ed. Engl. *50*, 10608–10611.
25. Rhee, S.G., Jeong, W., Chang, T.S., and Woo, H.A. (2007). Sulfiredoxin, the cysteine sulfinic acid reductase specific to 2-Cys peroxiredoxin: its discovery, mechanism of action, and biological significance. Kidney Int. Suppl. *72*, S3–S8.
26. O'Neill, J.S., and Reddy, A.B. (2011). Circadian clocks in human red blood cells. Nature *469*, 498–503.
27. Cho, C.S., Yoon, H.J., Kim, J.Y., Woo, H.A., and Rhee, S.G. (2014). Circadian rhythm of hyperoxidized peroxiredoxin II is determined by hemoglobin autoxidation and the 20S proteasome in red blood cells. Proc. Natl. Acad. Sci. USA *111*, 12043–12048.
28. Haraldsen, J.D., Liu, G., Botting, C.H., Walton, J.G., Storm, J., Phalen, T.J., Kwok, L.Y., Soldati-Favre, D., Heintz, N.H., Müller, S., *et al*. (2009). Identification of Conoidin a as a Covalent Inhibitor of Peroxiredoxin Ii. Org. Biomol. Chem. *7*, 3040–3048.
29. Zhang, L., Hastings, M.H., Green, E.W., Tauber, E., Sladek, M., Webster, S.G., Kyriacou, C.P., and Wilcockson, D.C. (2013). Dissociation of circadian and circatidal timekeeping in the marine crustacean Eurydice pulchra. Curr. Biol. *23*, 1863–1873.
30. Wolf, Y.I., and Koonin, E.V. (2013). Genome reduction as the dominant mode of evolution. BioEssays *35*, 829–837.
31. Ko, C.H., Yamada, Y.R., Welsh, D.K., Buhr, E.D., Liu, A.C., Zhang, E.E., Ralph, M.R., Kay, S.A., Forger, D.B., and Takahashi, J.S. (2010). Emergence of noise-induced oscillations in the central circadian pacemaker. PLoS Biol. *8*, e1000513.
32. Koumandou, V.L., Wickstead, B., Ginger, M.L., van der Giezen, M., Dacks, J.B., and Field, M.C. (2013). Molecular paleontology and complexity in the last eukaryotic common ancestor. Crit. Rev. Biochem. Mol. Biol. *48*, 373–396.
33. Stincone, A., Prigione, A., Cramer, T., Wamelink, M.M., Campbell, K., Cheung, E., Olin-Sandoval, V., Grüning, N., Krüger, A., Tauqeer Alam, M., *et al*. (2014). The return of metabolism: biochemistry and physiology of the pentose phosphate pathway. Biol. Rev. Camb. Philos. Soc. http://dx.doi.org/10.1111/brv.12140.
34. van Dijken, J.P., Bauer, J., Brambilla, L., Duboc, P., Francois, J.M., Gancedo, C., Giuseppin, M.L., Heijnen, J.J., Hoare, M., Lange, H.C., Madden, E.A., *et al*. (2000). An interlaboratory comparison of physiological and genetic properties of four Saccharomyces cerevisiae strains. Enzyme Microb. Technol. *26*, 706–714.
35. MacDiarmid, C.W., Taggart, J., Kerdsomboon, K., Kubisiak, M., Panascharoen, S., Schelble, K., and Eide, D.J. (2013). Peroxiredoxin chaperone activity is critical for protein homeostasis in zinc-deficient yeast. J. Biol. Chem. *288*, 31313–31327.
36. Robertson, J.B., and Johnson, C.H. (2011). Luminescence as a continuous real-time reporter of promoter activity in yeast undergoing respiratory oscillations or cell division rhythms. Methods Mol. Biol. *734*, 63–79.
37. Yoo, S.H., Yamazaki, S., Lowrey, P.L., Shimomura, K., Ko, C.H., Buhr, E.D., Siepka, S.M., Hong, H.K., Oh, W.J., Yoo, O.J., *et al*. (2004). PERIOD2:LUCIFERASE real-time reporting of circadian dynamics reveals persistent circadian oscillations in mouse peripheral tissues. Proc. Natl. Acad. Sci. USA *101*, 5339–5346.
38. O'Neill, J.S., and Hastings, M.H. (2008). Increased coherence of circadian rhythms in mature fibroblast cultures. J. Biol. Rhythms *23*, 483–488.
39. Xu, J. (2005). Preparation, culture, and immortalization of mouse embryonic fibroblasts. Curr. Protoc. Mol. Biol. *Chapter 28*, 1.
40. Hoeflich, K.P., Luo, J., Rubie, E.A., Tsao, M.S., Jin, O., and Woodgett, J.R. (2000). Requirement for glycogen synthase kinase-3beta in cell survival and NF-kappaB activation. Nature *406*, 86–90.

Current Biology

Supplemental Information

Metabolic Cycles in Yeast

Share Features Conserved among Circadian Rhythms

Helen C. Causton, Kevin A. Feeney, Christine A. Ziegler, and John O'Neill

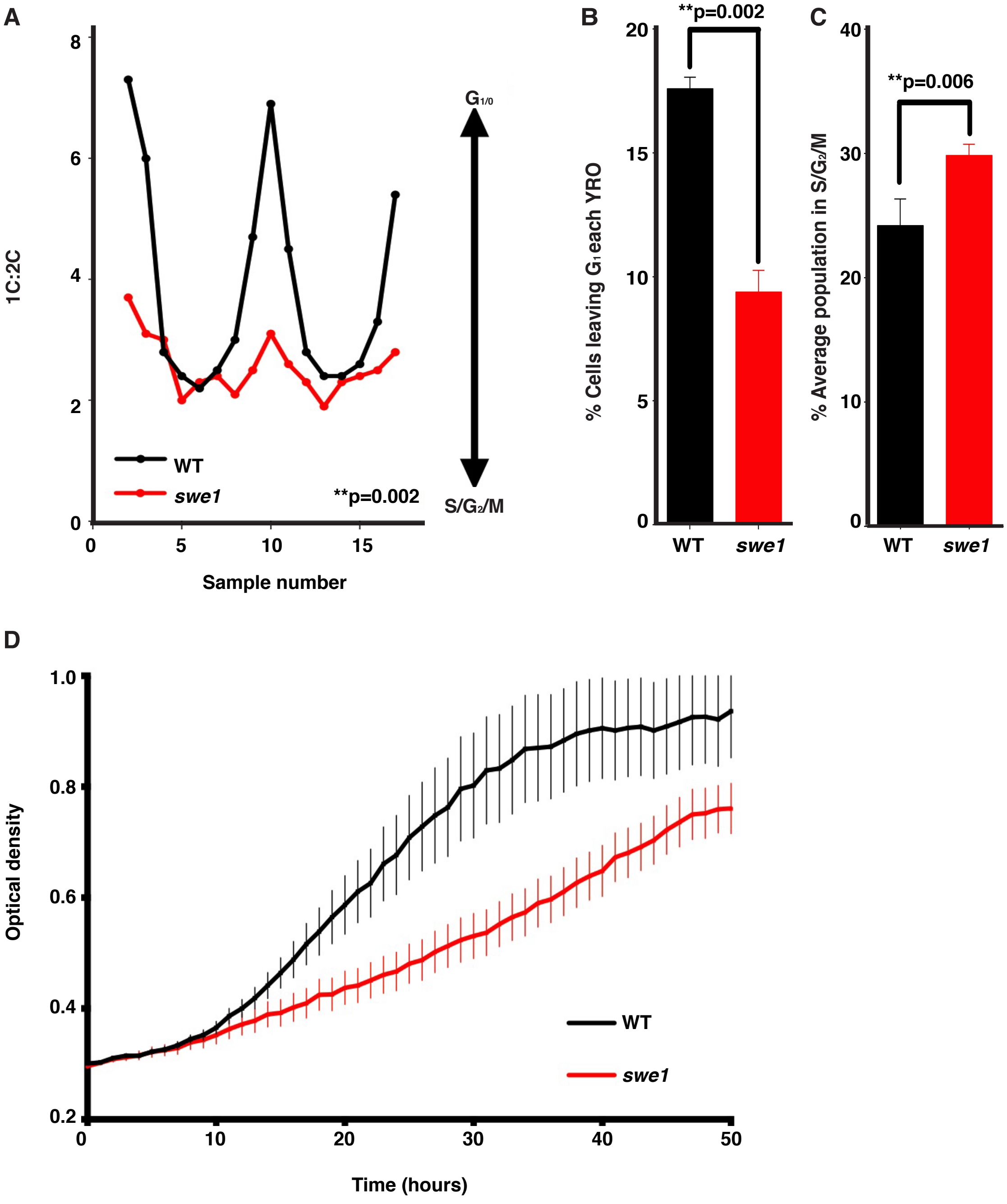

Figure S1, relating to Figure 1. Strains deleted for *SWE1* show less variation in DNA content across the YRO, leave G_1 less frequently and grow slower than wild type under YRO/bioreactor and standard growth conditions (**A**) Samples harvested across two oscillations were analysed for DNA content by propidium iodide staining and FACs analysis. Paired t-test, p=0.002. (**B**) Bar graph showing fewer *swe1* cells leave G_1 per respiratory oscillation when growing in the bioreactor. Paired t-test, p=0.002. (**C**) Bar graph showing that, on average, more cells are in the S/G2 or M phases of the cell cycle in *swe1* strains than in wild type under YRO/bioreactor conditions. Paired t-test, p=0.006. (**D**) Growth curves for wild type and *swe1* strains under standard growth conditions. Mean±SEM, n=4; 2-way ANOVA, p<0.0001 for time vs. genotype interaction.

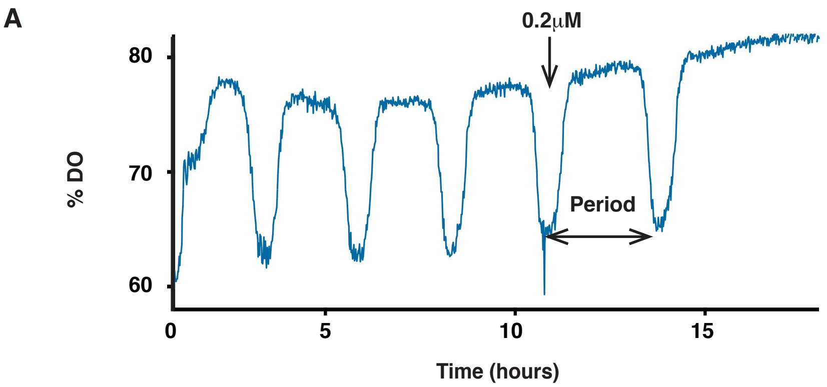

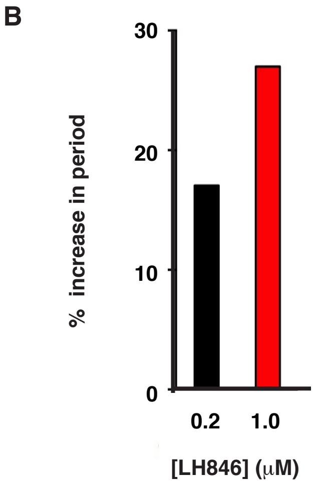

Figure S2, relating to Figure 2. Casein kinase inhibitor LH846 has a dose-dependent effect on the period of the YRO. (**A**) Representative dissolved oxygen trace indicating the time of addition of LH846. (**B**) Bar graph showing increase in period of oscillation in the presence of drug. Results from two independent experiments.

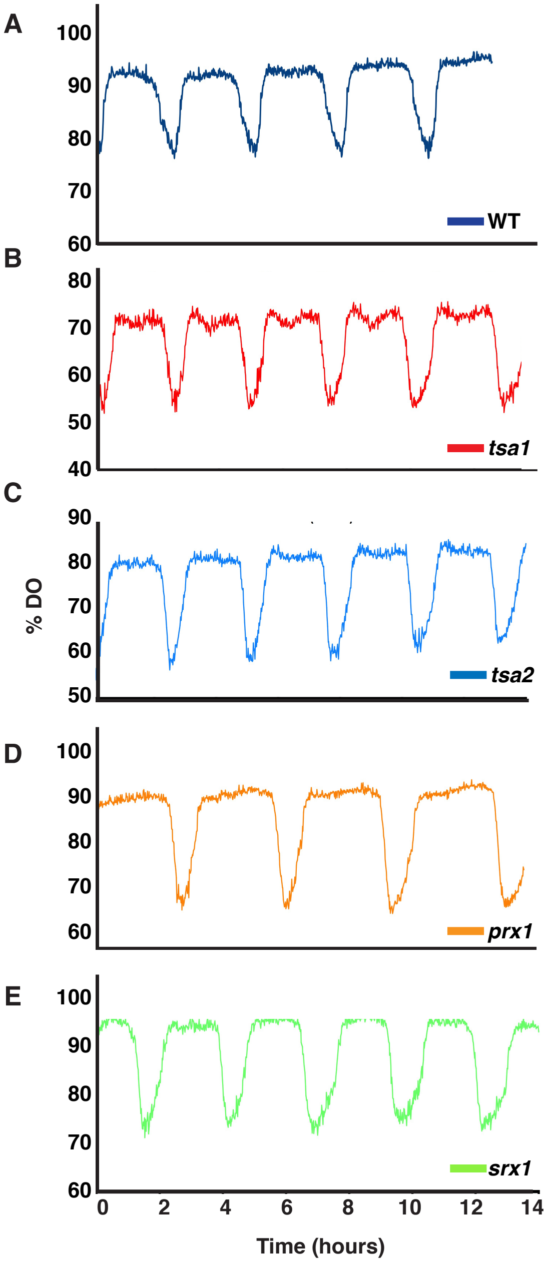

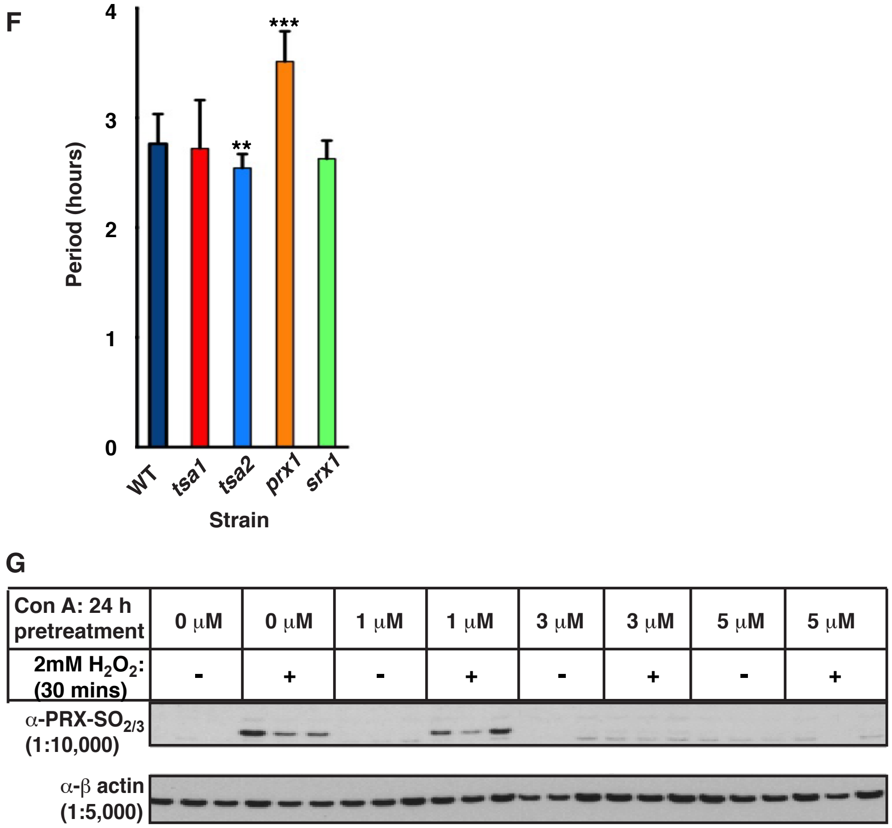

Figure S3, relating to Figure 4. Individual peroxiredoxins are not required for YROs and Conoidin A acts to block over-oxidation of cellular PRX in mouse fibroblasts treated with peroxide. (**A-E**) Representative dissolved oxygen trace for wild type and mutant strains. (**F**) Periods are wild type 2.77 h, SD 0.26, n=17; *tsa1* 2.72h, SD 0.44, n= 7, p=0.807; *tsa2* 2.53h, SD 0.13, n= 9, p=0.009; *prx1* 3.50h, SD 0.27, n= 5, p=0.001; *srx1* 2.62h, SD 0.17, n= 6, p=0.162. Mean ± SEM, p-values are t-test vs. wild type. (**G**) Western blot showing dose-dependent inhibition of PRX over-oxidation by 30 minutes 2 mM H_2O_2 treatment, following 24-hour pre-treatment with varying concentrations of conoidin A. SDS-PAGE was performed under reducing conditions, and performed as [S16] except that cells were pre-treated with 20 mM NEM in ice cold PBS for 15 minutes prior to lysis to alkylate any free thiol groups. Three biological replicates are shown per condition.

	Mammalian Cellular Circadian Rhythm	Yeast Respiratory Oscillation
**Cell autonomous, persists under constant conditions**	• Mouse fibroblasts and neurons [S1-S3].	• Period is consistent within a given strain/set of conditions [S4, S5-S10].
**Temperature compensated**	• In mouse fibroblasts [S11, S12].	• In *Schizosaccharomyces pombe* and *Saccharomyces cerevisiae* [S7, S13, S14].
**Involves redox/metabolic cycles**	• In human & mouse erythrocytes [S15, S16], mouse fibroblasts [S17, S18] and mouse myoblasts [S19].<br>• Mitochondrial metabolism [S19].<br>• Glucose utilization [S20].	• Redox balance<br>NAD(P)H [S6, S8, S21, S22-S25].<br>Glutathione and GSH [S5, S21, S24]<br>• Mitochondrial metabolism [S6, S23, S26, S27].<br>• Glucose utilization [S8, S9, S28].
**Involves rhythmic transcription and chromatin modification**	• In mouse fibroblasts [S29, S30-S32].	• Transcription [S6, S9, S33, S34].<br>• Histone acetylation [S35].<br>• Chromatin modification [S36].
**Involves temporal regulation of haem and carbon monoxide**	• Agonists of clock gene REV-ERB's haem binding site resets clock in mouse fibroblasts [S37].<br>• Haem synthesis is circadian regulated [S38].<br>• Other clock genes reported as functionally regulated by haem [S39, S40].	• The concentration of aminolevulinic acid, a rate-limiting metabolite in the synthesis of haem, oscillates [S24].<br>• Addition of CO can induce phase advancement, strains deleted for haem oxygenase oscillate with a longer period [S41].
**Coupled with DNA replication and cell division**	• In mouse fibroblasts [S2, S42-S44].	• DNA replication is gated to the reductive phase of the cycle [S6, S9, S33]. This may function to reduce the mutation rate [S7, S45].<br>• Oscillations are typically sychronised with the budding index [S25, S46, S47].<br>• Respiratory oscillations can occur without cell division cycling [S48].<br>• Swe1 is required for entry to the cell cycle after G_1 arrest [S49] and *SWE1* expression varies more than 10 fold across the YRO [S6].

Table S1.
Note that the mammalian references pertain specifically to observations made using cultured mammalian cells *in vitro* so as to be comparable with the yeast references.

		Casein kinase 1 (CK1)	Glycogen synthase kinase 3 (GSK3)
	Description	• Family of nucleocytoplasmic Ser/Thr kinases<br>• Highly conserved in eukaryotes, ubiquitously expressed<br>• Auto-phosphorylation inactivates<br>• Usually requires a priming phosphate (pS/T-X-X-S)<br>• Can act synergistically with other kinases, such as GSK3 and PKA, at multisite phosphorylation domains to regulate protein stability and nucleo-cytoplasmic shuttling [S50, S51].	• Family of nucleocytoplasmic Ser/Thr kinases<br>• Highly conserved in eukaryotes, ubiquitously expressed<br>• Akt/PKB phosphorylation inactivates<br>• Usually requires a priming phosphate (S/T-X-X-X-pS)<br>• Can act synergistically with other kinases, such as CK1 and CK2, at multisite phosphorylation domains to regulate protein stability and nucleo-cytoplasmic shuttling [S50, S52].
**Cellular functions (non-circadian)**	Mammals	• 5 isoforms (7 genes) in mammals (α, β, γ1-3, δ, ε) with multiple cellular substrates and partners.<br>• Important roles in membrane trafficking, DNA replication/repair, cytokinesis, vesicular transport, ribosome biogenesis, and transcription [S53, S54].<br>• Several CK1 isoforms work co-ordinately regulate wnt signalling [S55].<br>• Targets proteins such as β-catenin and IκB for ubiquitin-mediated proteasomal degradation through recruitment of F-box proteins (e.g. β-TRCP) that recognise the phosphodegron (Dp-S-G-X-XpS) [S56].	• 2 isoforms in mammals (α/β) with multiple cellular substrates and partners [S52].<br>• Phosphorylates enzymes such as glycogen synthase and IRS1 to regulate glucose homeostasis [S57, S58].<br>• Essential role in development (part of canonical β-catenin/wnt signalling pathway) [S59].<br>• Regulates apoptosis, cell proliferation and migration [S60].<br>• Important signalling role in both the adaptive and innate immune responses [S61].<br>• Regulates cellular response to DNA damage e.g. through phosphorylation of p53 [S62].
	Flies	• 8 family members with diverse roles in cytoskeletal polarisation/morphogenesis [S63], glial cell migration [S64], olfactory learning [S65], sperm individualization [S66], regulator of Wnt and Hedgehog signaling [S67].<br>• Synergystic regulation of β-catenin/Armadillo with GSK3, similarly to mammalian cells [S68].	• Fly ortholog (*Shaggy, Sgg*) is a segment polarity gene best known as a repressor of Wingless (Wg) signalling [S69], but also required for normal growth of larval and imaginal tissues [S70].<br>• Also involved in attachment of the mitotic spindle at the cell cortex [S71], and repression of Hedgehog signaling where it acts synergistically with CK1 to regulate protein degradation [S72].
	Plants	• Plants encode dozens of CK1 homologs, mostly uncharacterised. Roles include subcellular targeting [S73], root development, plant hormone sensitivity [S74], microtubule organization [S75] and starch metabolism [S76].	• The large family of GSK3 homologs in plants are implicated in diverse roles such as regulation of vascular development [S77], abscisic acid signalling [S78], floral development [S79], cell growth/differentiation [S80].<br>• GSK3-like kinase BIN3 phosphorylates transcription factor BIN2, to stabilise it and thereby negatively regulate brassinosteroid signalling [S81].
	Fungi (*S. cerevisiae*)	• 4 CK1 isoforms in *S. cerevisae*: – Yck1/2 functions in ROS signalling, to directly integrate signals from oxygen and glucose and repress transcription [S82].<br>• CK1δ homolog, Hrr25 has multiple functions including repression of calcineurin signalling [S83].	• Yeast homolog, Rim11, part of a feed forward loop that switches diploid yeast from a proliferative cycle to the meiotic cycle in response to nutrients [S84].<br>• Acts with PKA to regulate the stability of the stress-responsive transcription factor Cin5 [S85].
**Circadian clock function**	Mammals	• The gain-of-function tau mutation in CK1ε shortens circadian period by ~3 hours in homozygotes [S86-S88].<br>• Human familial sleep disorders (early awakening) segregate with mutations in human CK1δ or hPER2 phosphorylation sites [S89, S90].<br>• CK1δ/ε determine PER1/2 protein degradation, complex formation and nuclear import/export in the absence of both enzymes, the canonical transcriptional oscillator stops completely [S91, S92]. CK1α also recently implicated [S93].	• GSK3 exhibits a cell-autonomous phosphorylation rhythm [S94, S95].<br>• Knockdown/pharmacological inhibition of GSK3β shortens circadian period [S96]. Constitutive activation lengthens circadian period [S94].<br>• Phosphorylates clock proteins BMAL1, CLOCK, CRY2, PER2 and REV-ERBα to regulate their stability [S97, S98, S99].
	Flies	• Early mutagenesis screens identified CK1δ/ε homolog (*doubletime, dbt*), which regulates period length through phosphorylation of dPER [S100].<br>• Phosphorylation determines dPER turnover kinetics, complex formation and nucleocytoplasmic shuttling [S100, S101].	• GSK3β homolog (*shaggy, sgg*) was originally implicated in timekeeping in Drosophila mutants that exhibit altered circadian period [S102].<br>• GSK3 phosphorylates components of the core clock machinery (*e.g.*, TIMELESS) to regulate clock complex stability, localisation and periodicity [S103].
	Plants/ algae	• CK1 over-expression or inhibition lengthens circadian period in *O. tauri* ([S104, S105].<br>• CK1 homologs phosphorylate Cryptochrome (blue light sensor) in *O. tauri* and *A. thaliana* [S105, S106].	• Pharmacological inhibitors of GSK3 shorten circadian period dose-dependently in *O. tauri* [S104].<br>• Clock-relevant targets are not known at present.
	Fungi (*N. Crassa*)	• A CK1δ/ε homolog phosphorylates FRQ and WC1/2 to regulate the kinetics of protein turnover, thereby determining circadian period length [S107, S108, S109].	• A GSK3 homolog phosphorylates WC-1/2 to regulate protein abundance, with knockdown leading to WC-1 accumulation and shortened circadian period [S110].

Table S2. A comparison between the cellular and circadian functions of CK1 and GSK3 in model eukaryotes.

All the strains are isogenic and were made in the prototrophic CEN.PK background [S111].

HCY1377	*Mata TSA1::KanMX*
HCY1381	*Mata TSA2::KanMX*
HCY1385	*Mata PRX1::KanMX*
HCY1414	*Mata SRX1::KanMX*
HCY1427	*Mata TSA1::NatMX TSA2::KanMX*
HCY1445	*Mata RIM11::KanMX*
HCY1465	*Mata SWE1::KanMX*

Table S3. Strains and genotypes

Supplemental References

S1. Welsh, D.K., Yoo, S.H., Liu, A.C., Takahashi, J.S., and Kay, S.A. (2004). Bioluminescence imaging of individual fibroblasts reveals persistent, independently phased circadian rhythms of clock gene expression. Curr Biol *14*, 2289-2295.

S2. Nagoshi, E., Saini, C., Bauer, C., Laroche, T., Naef, F., and Schibler, U. (2004). Circadian gene expression in individual fibroblasts: cell-autonomous and self-sustained oscillators pass time to daughter cells. Cell *119*, 693-705.

S3. Welsh, D.K., Logothetis, D.E., Meister, M., and Reppert, S.M. (1995). Individual neurons dissociated from rat suprachiasmatic nucleus express independently phased circadian firing rhythms. Neuron *14*, 697-706.

S4. Keulers, M., Satroutdinov, A.D., Suzuki, T., and Kuriyama, H. (1996). Synchronization affector of autonomous short-period-sustained oscillation of Saccharomyces cerevisiae. Yeast *12*, 673-682.

S5. Sohn, H.-Y., and Kuriyama, H. (2001). Ultradian metabolic oscillation of *Saccharomyces cerevisiae* during aerobic continuous culture: hydrogen sulphide, a population synchronizer, is produced by sulphite reductase. Yeast *18*, 125-135.

S6. Tu, B.P., Kudlicki, A., Rowicka, M., and McKnight, S.L. (2005). Logic of the yeast metabolic cycle: temporal compartmentalization of cellular processes. Science *310*, 1152-1158.

S7. Chen, Z., and McKnight, S.L. (2007). A conserved DNA damage response pathway responsible for coupling the cell division cycle to the circadian and metabolic cycles. Cell Cycle *6*, 2906-2912.

S8. Ouyang, Y., Xu, Q., Mitsui, K., Motizuki, M., and Xu, Z. (2011). PSK2 coordinates glucose metabolism and utilization to maintain ultradian clock-coupled respiratory oscillation in Saccharomyces cerevisiae yeast. Archives of biochemistry and biophysics *509*, 52-58.

S9. Chin, S.L., Marcus, I.M., Klevecz, R.R., and Li, C.M. (2012). Dynamics of oscillatory phenotypes in Saccharomyces cerevisiae reveal a network of genome-wide transcriptional oscillators. The FEBS journal *279*, 1119-1130.

S10. Eelderink-Chen, Z., Mazzotta, G., Sturre, M., Bosman, J., Roenneberg, T., and Merrow, M. (2010). A circadian clock in Saccharomyces cerevisiae. Proc Natl Acad Sci U S A *107*, 2043-2047.

S11. Tsuchiya, Y., Akashi, M., and Nishida, E. (2003). Temperature compensation and temperature resetting of circadian rhythms in mammalian cultured fibroblasts. Genes to cells : devoted to molecular & cellular mechanisms *8*, 713-720.

S12. Izumo, M., Johnson, C.H., and Yamazaki, S. (2003). Circadian gene expression in mammalian fibroblasts revealed by real-time luminescence reporting: temperature compensation and damping. Proc Natl Acad Sci U S A *100*, 16089-16094.

S13. Kippert, F., and Lloyd, D. (1995). A temperature-compensated ultradian clock ticks in Schizosaccharomyces pombe. Microbiology *141 (Pt 4)*, 883-890.

S14. Murray, D.B., Roller, S., Kuriyama, H., and Lloyd, D. (2001). Clock control of ultradian respiratory oscillation found during yeast continuous culture. Journal of bacteriology *183*, 7253-7259.

S15. Cho, C.S., Yoon, H.J., Kim, J.Y., Woo, H.A., and Rhee, S.G. (2014). Circadian rhythm of hyperoxidized peroxiredoxin II is determined by hemoglobin autoxidation and the 20S proteasome in red blood cells. Proc Natl Acad Sci U S A.

S16. Milev, N.B., Rey, G., Valekunja, U.K., Edgar, R.S., O'Neill, J.S., and Reddy, A.B. (2014). Analysis of the Redox Oscillations in the Circadian Clockwork. Methods in Enzymology *In press*, 617-642.

S17. O'Neill, J.S., and Reddy, A.B. (2011). Circadian clocks in human red blood cells. Nature *469*, 498-503.

S18. O'Neill, J.S., and Feeney, K.A. (2013). Circadian Redox and Metabolic Oscillations in Mammalian Systems. Antioxidants & redox signaling.

S19. Peek, C.B., Affinati, A.H., Ramsey, K.M., Kuo, H.Y., Yu, W., Sena, L.A., Ilkayeva, O., Marcheva, B., Kobayashi, Y., Omura, C., et al. (2013). Circadian clock NAD+ cycle drives mitochondrial oxidative metabolism in mice. Science *342*, 1243417.

S20. Paulose, J.K., Rucker, E.B., 3rd, and Cassone, V.M. (2012). Toward the beginning of time: circadian rhythms in metabolism precede rhythms in clock gene expression in mouse embryonic stem cells. PLoS One *7*, e49555.

S21. Murray, D.B., Engelen, F., Lloyd, D., and Kuriyama, H. (1999). Involvement of glutathione in the regulation of respiratory oscillation during a continuous culture of *Saccharomyces cerevisiae*. Microbiol. *145*, 2739-2745.

S22. Wang, J., Liu, W., Uno, T., Tonozuka, H., Mitsui, K., and Tsurugi, K. (2000). Cellular stress responses oscillate in synchronization with the ultradian oscillation of energy metabolism in the yeast *Saccharomyces cerevisiae*. FEMS Microbiol. Lett. *189*, 9-13.

S23. Lloyd, D., Salgado, L.E., Turner, M.P., Suller, M.T., and Murray, D. (2002). Cycles of mitochondrial energization driven by the ultradian clock in a continuous culture of Saccharomyces cerevisiae. Microbiology *148*, 3715-3724.

S24. Tu, B.P., Mohler, R.E., Liu, J.C., Dombek, K.M., Young, E.T., Synovec, R.E., and McKnight, S.L. (2007). Cyclic changes in metabolic state during the life of a yeast cell. P.N.A.S (USA) *104*, 16886-16891.

S25. Xu, Z., and Tsurugi, K. (2007). Destabilization of energy-metabolism oscillation in the absence of trehalose synthesis in the chemostat culture of yeast. Archives of biochemistry and biophysics *464*, 350-358.

S26. Kwak, W.J., Kwon, G.S., Jin, I., Kuriyama, H., and Sohn, H.Y. (2003). Involvement of oxidative stress in the regulation of H(2)S production during ultradian metabolic oscillation of Saccharomyces cerevisiae. FEMS Microbiol Lett *219*, 99-104.

S27. Sohn, H.-Y., Kum, E.-J., Kwon, G.-S., Jin, I., and Kuriyama, H. (2005). Regulation of branched-chain, and sulfur-containing amino acid metabolism by glutathione during ultradian metabolic oscillation of *Saccharomyces cerevisiae*. J. Microbiol. *43*, 375-380.

S28. Wittmann, C., Hans, M., van Winden, W.A., Ras, C., and Heijnen, J.J. (2005). Dynamics of intracellular metabolites of glycolysis and TCA cycle during cell-cycle-related oscillation in Saccharomyces cerevisiae. Biotechnology and bioengineering *89*, 839-847.

S29. Naruse, Y., Oh-hashi, K., Iijima, N., Naruse, M., Yoshioka, H., and Tanaka, M. (2004). Circadian and light-induced transcription of clock gene Per1 depends on histone acetylation and deacetylation. Mol Cell Biol *24*, 6278-6287.

S30. Fogg, P.C., O'Neill, J.S., Dobrzycki, T., Calvert, S., Lord, E.C., McIntosh, R.L., Elliott, C.J., Sweeney, S.T., Hastings, M.H., and Chawla, S. (2014). Class IIa histone deacetylases are conserved regulators of circadian function. J Biol Chem.

S31. Nakahata, Y., Kaluzova, M., Grimaldi, B., Sahar, S., Hirayama, J., Chen, D., Guarente, L.P., and Sassone-Corsi, P. (2008). The NAD+-dependent deacetylase SIRT1 modulates CLOCK-mediated chromatin remodeling and circadian control. Cell *134*, 329-340.

S32. Aguilar-Arnal, L., Hakim, O., Patel, V.R., Baldi, P., Hager, G.L., and Sassone-Corsi, P. (2013). Cycles in spatial and temporal chromosomal organization driven by the circadian clock. Nature structural & molecular biology *20*, 1206-1213.

S33. Klevecz, R.R., Bolen, J., Forrest, G., and Murray, D.B. (2004). A genomewide oscillation in transcription gates DNA replication and cell cycle. P.N.A.S (USA) *101*, 1200-1205.

S34. Li, C.M., and Klevecz, R.R. (2006). A rapid genome-scale response of the transcriptional oscillator to perturbation reveals a period-doubling path to phenotypic change. Proc Natl Acad Sci U S A *103*, 16254-16259.

S35. Cai, L., Sutter, B.M., Li, B., and Tu, B.P. (2011). Acetyl-CoA induces cell growth and proliferation by promoting the acetylation of histones at growth genes. Mol Cell *42*, 426-437.

S36. Machné, R., and Murray, D.B. (2012). The yin and yang of yeast transcription: elements of a global feedback system between metabolism and chromatin. PLoS One *7*, e37906.

S37. Meng, Q.J., McMaster, A., Beesley, S., Lu, W.Q., Gibbs, J., Parks, D., Collins, J., Farrow, S., Donn, R., Ray, D., et al. (2008). Ligand modulation of REV-ERBalpha function resets the peripheral circadian clock in a phasic manner. J Cell Sci *121*, 3629-3635.

S38. Kaasik, K., and Lee, C.C. (2004). Reciprocal regulation of haem biosynthesis and the circadian clock in mammals. Nature *430*, 467-471.

S39. Dioum, E.M., Rutter, J., Tuckerman, J.R., Gonzalez, G., Gilles-Gonzalez, M.A., and McKnight, S.L. (2002). NPAS2: a gas-responsive transcription factor. Science *298*, 2385-2387.

S40. Yin, L., Wu, N., Curtin, J.C., Qatanani, M., Szwergold, N.R., Reid, R.A., Waitt, G.M., Parks, D.J., Pearce, K.H., Wisely, G.B., et al. (2007). Rev-erbalpha, a heme sensor that coordinates metabolic and circadian pathways. Science *318*, 1786-1789.

S41. Tu, B.P., and McKnight, S.L. (2009). Evidence of carbon monoxide-mediated phase advancement of the yeast metabolic cycle. Proc Natl Acad Sci U S A *106*, 14293-14296.

S42. Matsuo, T., Yamaguchi, S., Mitsui, S., Emi, A., Shimoda, F., and Okamura, H. (2003). Control mechanism of the circadian clock for timing of cell division in vivo. Science *302*, 255-259.

S43. Bieler, J., Cannavo, R., Gustafson, K., Gobet, C., Gatfield, D., and Naef, F. (2014). Robust synchronization of coupled circadian and cell cycle oscillators in single mammalian cells. Mol Syst Biol *10*, 739.

S44. Feillet, C., Krusche, P., Tamanini, F., Janssens, R.C., Downey, M.J., Martin, P., Teboul, M., Saito, S., Levi, F.A., Bretschneider, T., et al. (2014). Phase locking and multiple oscillating attractors for the coupled mammalian clock and cell cycle. Proc Natl Acad Sci U S A *111*, 9828-9833.

S45. Chen, Z., Odstrcil, E.A., Tu, B.P., and McKnight, S.L. (2007). Restriction of DNA replication to the reductive phase of the metabolic cycle protects genome integrity. Science *316*, 1916-1919.

S46. Porro, D., Martegani, E., Ranzi, B.M., and Alberghina, L. (1988). Oscillations in continuous cultures of budding yeast: a segregated parameter analysis. Biotechnology and bioengineering *32*, 411-417.

S47. Slavov, N., and Botstein, D. (2011). Coupling among growth rate response, metabolic cycle, and cell division cycle in yeast. Mol Biol Cell *22*, 1997-2009.

S48. **Slavov, N., Macinskas, J., Caudy, A., and Botstein, D. (2011). Metabolic cycling without cell division cycling in respiring yeast. Proc Natl Acad Sci U S A *108*, 19090-19095.**
S49. **Saracino, F., Bassler, J., Muzzini, D., Hurt, E., and Agostoni Carbone, M.L. (2004). The yeast kinase Swe1 is required for proper entry into cell cycle after arrest due to ribosome biogenesis and protein synthesis defects. Cell Cycle *3*, 648-654.**
S50. **Rena, G., Bain, J., Elliott, M., and Cohen, P. (2004). D4476, a cell-permeant inhibitor of CK1, suppresses the site-specific phosphorylation and nuclear exclusion of FOXO1a. EMBO reports *5*, 60-65.**
S51. **Hunter, T. (2007). The age of crosstalk: phosphorylation, ubiquitination, and beyond. Mol Cell *28*, 730-738.**
S52. **Xu, C., Kim, N.G., and Gumbiner, B.M. (2009). Regulation of protein stability by GSK3 mediated phosphorylation. Cell Cycle *8*, 4032-4039.**
S53. **Cheong, J.K., and Virshup, D.M. (2011). Casein kinase 1: Complexity in the family. The international journal of biochemistry & cell biology *43*, 465-469.**
S54. **Schittek, B., and Sinnberg, T. (2014). Biological functions of casein kinase 1 isoforms and putative roles in tumorigenesis. Molecular cancer *13*, 231.**
S55. **Del Valle-Perez, B., Arques, O., Vinyoles, M., de Herreros, A.G., and Dunach, M. (2011). Coordinated action of CK1 isoforms in canonical Wnt signaling. Mol Cell Biol *31*, 2877-2888.**
S56. **Zhao, B., Li, L., Tumaneng, K., Wang, C.Y., and Guan, K.L. (2010). A coordinated phosphorylation by Lats and CK1 regulates YAP stability through SCF(beta-TRCP). Genes Dev *24*, 72-85.**
S57. **Embi, N., Rylatt, D.B., and Cohen, P. (1980). Glycogen synthase kinase-3 from rabbit skeletal muscle. Separation from cyclic-AMP-dependent protein kinase and phosphorylase kinase. Eur J Biochem *107*, 519-527.**
S58. **Liberman, Z., and Eldar-Finkelman, H. (2005). Serine 332 phosphorylation of insulin receptor substrate-1 by glycogen synthase kinase-3 attenuates insulin signaling. J Biol Chem *280*, 4422-4428.**
S59. **Metcalfe, C., and Bienz, M. (2011). Inhibition of GSK3 by Wnt signalling--two contrasting models. J Cell Sci *124*, 3537-3544.**
S60. **Jope, R.S., and Johnson, G.V. (2004). The glamour and gloom of glycogen synthase kinase-3. Trends Biochem Sci *29*, 95-102.**
S61. **Wang, H., Brown, J., and Martin, M. (2011). Glycogen synthase kinase 3: a point of convergence for the host inflammatory response. Cytokine *53*, 130-140.**
S62. **Watcharasit, P., Bijur, G.N., Zmijewski, J.W., Song, L., Zmijewska, A., Chen, X., Johnson, G.V., and Jope, R.S. (2002). Direct, activating interaction between glycogen synthase kinase-3beta and p53 after DNA damage. Proc Natl Acad Sci U S A *99*, 7951-7955.**
S63. **Gault, W.J., Olguin, P., Weber, U., and Mlodzik, M. (2012). Drosophila CK1-gamma, gilgamesh, controls PCP-mediated morphogenesis through regulation of vesicle trafficking. J Cell Biol *196*, 605-621.**
S64. **Hummel, T., Attix, S., Gunning, D., and Zipursky, S.L. (2002). Temporal control of glial cell migration in the Drosophila eye requires gilgamesh, hedgehog, and eye specification genes. Neuron *33*, 193-203.**
S65. **Tan, Y., Yu, D., Pletting, J., and Davis, R.L. (2010). Gilgamesh is required for rutabaga-independent olfactory learning in Drosophila. Neuron *67*, 810-820.**
S66. **Nerusheva, O.O., Dorogova, N.V., Gubanova, N.V., Yudina, O.S., and Omelyanchuk, L.V. (2009). A GFP trap study uncovers the functions of Gilgamesh protein kinase in Drosophila melanogaster spermatogenesis. Cell biology international *33*, 586-593.**

S67. **Price, M.A. (2006). CKI, there's more than one: casein kinase I family members in Wnt and Hedgehog signaling. Genes Dev *20*, 399-410.**

S68. **Cadigan, K.M., and Peifer, M. (2009). Wnt signaling from development to disease: insights from model systems. Cold Spring Harbor perspectives in biology *1*, a002881.**

S69. **Siegfried, E., Chou, T.B., and Perrimon, N. (1992). wingless signaling acts through zeste-white 3, the Drosophila homolog of glycogen synthase kinase-3, to regulate engrailed and establish cell fate. Cell *71*, 1167-1179.**

S70. **Bourouis, M., Heitzler, P., el Messal, M., and Simpson, P. (1989). Mutant Drosophila embryos in which all cells adopt a neural fate. Nature *341*, 442-444.**

S71. **McCartney, B.M., McEwen, D.G., Grevengoed, E., Maddox, P., Bejsovec, A., and Peifer, M. (2001). Drosophila APC2 and Armadillo participate in tethering mitotic spindles to cortical actin. Nature cell biology *3*, 933-938.**

S72. **Price, M.A., and Kalderon, D. (2002). Proteolysis of the Hedgehog signaling effector Cubitus interruptus requires phosphorylation by Glycogen Synthase Kinase 3 and Casein Kinase 1. Cell *108*, 823-835.**

S73. **Lee, J.Y. (2009). Versatile casein kinase 1: multiple locations and functions. Plant signaling & behavior *4*, 652-654.**

S74. **Liu, W., Xu, Z.H., Luo, D., and Xue, H.W. (2003). Roles of OsCKI1, a rice casein kinase I, in root development and plant hormone sensitivity. The Plant journal : for cell and molecular biology *36*, 189-202.**

S75. **Ben-Nissan, G., Cui, W., Kim, D.J., Yang, Y., Yoo, B.C., and Lee, J.Y. (2008). Arabidopsis casein kinase 1-like 6 contains a microtubule-binding domain and affects the organization of cortical microtubules. Plant physiology *148*, 1897-1907.**

S76. **Min, L., Zhu, L., Tu, L., Deng, F., Yuan, D., and Zhang, X. (2013). Cotton GhCKI disrupts normal male reproduction by delaying tapetum programmed cell death via inactivating starch synthase. The Plant journal : for cell and molecular biology *75*, 823-835.**

S77. **Kondo, Y., Fujita, T., Sugiyama, M., and Fukuda, H. (2014). A novel system for xylem cell differentiation in Arabidopsis thaliana. Molecular plant.**

S78. **Cai, Z., Liu, J., Wang, H., Yang, C., Chen, Y., Li, Y., Pan, S., Dong, R., Tang, G., Barajas-Lopez Jde, D., et al. (2014). GSK3-like kinases positively modulate abscisic acid signaling through phosphorylating subgroup III SnRK2s in Arabidopsis. Proc Natl Acad Sci U S A *111*, 9651-9656.**

S79. **Qi, X., Chanderbali, A.S., Wong, G.K., Soltis, D.E., and Soltis, P.S. (2013). Phylogeny and evolutionary history of glycogen synthase kinase 3/SHAGGY-like kinase genes in land plants. BMC evolutionary biology *13*, 143.**

S80. **Youn, J.H., Kim, T.W., Kim, E.J., Bu, S., Kim, S.K., Wang, Z.Y., and Kim, T.W. (2013). Structural and functional characterization of Arabidopsis GSK3-like kinase AtSK12. Molecules and cells *36*, 564-570.**

S81. **Zhang, D., Ye, H., Guo, H., Johnson, A., Zhang, M., Lin, H., and Yin, Y. (2014). Transcription factor HAT1 is phosphorylated by BIN2 kinase and mediates brassinosteroid repressed gene expression in Arabidopsis. The Plant journal : for cell and molecular biology *77*, 59-70.**

S82. **Reddi, A.R., and Culotta, V.C. (2013). SOD1 integrates signals from oxygen and glucose to repress respiration. Cell *152*, 224-235.**

S83. **Kafadar, K.A., Zhu, H., Snyder, M., and Cyert, M.S. (2003). Negative regulation of calcineurin signaling by Hrr25p, a yeast homolog of casein kinase I. Genes Dev *17*, 2698-2708.**

S84. Rubin-Bejerano, I., Sagee, S., Friedman, O., Pnueli, L., and Kassir, Y. (2004). The in vivo activity of Ime1, the key transcriptional activator of meiosis-specific genes in Saccharomyces cerevisiae, is inhibited by the cyclic AMP/protein kinase A signal pathway through the glycogen synthase kinase 3-beta homolog Rim11. Mol Cell Biol *24*, 6967-6979.

S85. Pereira, J., Pimentel, C., Amaral, C., Menezes, R.A., and Rodrigues-Pousada, C. (2009). Yap4 PKA- and GSK3-dependent phosphorylation affects its stability but not its nuclear localization. Yeast *26*, 641-653.

S86. Ralph, M.R., and Menaker, M. (1988). A mutation of the circadian system in golden hamsters. Science *241*, 1225-1227.

S87. Lowrey, P.L., Shimomura, K., Antoch, M.P., Yamazaki, S., Zemenides, P.D., Ralph, M.R., Menaker, M., and Takahashi, J.S. (2000). Positional syntenic cloning and functional characterization of the mammalian circadian mutation tau. Science *288*, 483-492.

S88. Meng, Q.J., Logunova, L., Maywood, E.S., Gallego, M., Lebiecki, J., Brown, T.M., Sladek, M., Semikhodskii, A.S., Glossop, N.R., Piggins, H.D., et al. (2008). Setting clock speed in mammals: the CK1 epsilon tau mutation in mice accelerates circadian pacemakers by selectively destabilizing PERIOD proteins. Neuron *58*, 78-88.

S89. Xu, Y., Padiath, Q.S., Shapiro, R.E., Jones, C.R., Wu, S.C., Saigoh, N., Saigoh, K., Ptacek, L.J., and Fu, Y.H. (2005). Functional consequences of a CKIdelta mutation causing familial advanced sleep phase syndrome. Nature *434*, 640-644.

S90. Toh, K.L., Jones, C.R., He, Y., Eide, E.J., Hinz, W.A., Virshup, D.M., Ptacek, L.J., and Fu, Y.H. (2001). An hPer2 phosphorylation site mutation in familial advanced sleep phase syndrome. Science *291*, 1040-1043.

S91. Lee, H.M., Chen, R., Kim, H., Etchegaray, J.P., Weaver, D.R., and Lee, C. (2011). The period of the circadian oscillator is primarily determined by the balance between casein kinase 1 and protein phosphatase 1. Proc Natl Acad Sci U S A *108*, 16451-16456.

S92. Meng, Q.J., Maywood, E.S., Bechtold, D.A., Lu, W.Q., Li, J., Gibbs, J.E., Dupre, S.M., Chesham, J.E., Rajamohan, F., Knafels, J., et al. (2010). Entrainment of disrupted circadian behavior through inhibition of casein kinase 1 (CK1) enzymes. Proc Natl Acad Sci U S A *107*, 15240-15245.

S93. Hirota, T., Lee, J.W., Lewis, W.G., Zhang, E.E., Breton, G., Liu, X., Garcia, M., Peters, E.C., Etchegaray, J.P., Traver, D., et al. (2010). High-throughput chemical screen identifies a novel potent modulator of cellular circadian rhythms and reveals CKIalpha as a clock regulatory kinase. PLoS Biol *8*, e1000559.

S94. Paul, J.R., Johnson, R.L., Jope, R.S., and Gamble, K.L. (2012). Disruption of circadian rhythmicity and suprachiasmatic action potential frequency in a mouse model with constitutive activation of glycogen synthase kinase 3. Neuroscience *226*, 1-9.

S95. Iitaka, C., Miyazaki, K., Akaike, T., and Ishida, N. (2005). A role for glycogen synthase kinase-3beta in the mammalian circadian clock. J Biol Chem *280*, 29397-29402.

S96. Hirota, T., Lewis, W.G., Liu, A.C., Lee, J.W., Schultz, P.G., and Kay, S.A. (2008). A chemical biology approach reveals period shortening of the mammalian circadian clock by specific inhibition of GSK-3beta. Proc Natl Acad Sci U S A *105*, 20746-20751.

S97. Yin, L., Wang, J., Klein, P.S., and Lazar, M.A. (2006). Nuclear receptor Rev-erbalpha is a critical lithium-sensitive component of the circadian clock. Science *311*, 1002-1005.

S98. Sahar, S., Zocchi, L., Kinoshita, C., Borrelli, E., and Sassone-Corsi, P. (2010). Regulation of BMAL1 protein stability and circadian function by GSK3beta-mediated phosphorylation. PLoS One *5*, e8561.

S99. Spengler, M.L., Kuropatwinski, K.K., Schumer, M., and Antoch, M.P. (2009). A serine cluster mediates BMAL1-dependent CLOCK phosphorylation and degradation. Cell Cycle *8*, 4138-4146.

S100. Price, J.L., Blau, J., Rothenfluh, A., Abodeely, M., Kloss, B., and Young, M.W. (1998). double-time is a novel Drosophila clock gene that regulates PERIOD protein accumulation. Cell *94*, 83-95.

S101. Kloss, B., Price, J.L., Saez, L., Blau, J., Rothenfluh, A., Wesley, C.S., and Young, M.W. (1998). The Drosophila clock gene double-time encodes a protein closely related to human casein kinase Iepsilon. Cell *94*, 97-107.

S102. Martinek, S., Inonog, S., Manoukian, A.S., and Young, M.W. (2001). A role for the segment polarity gene shaggy/GSK-3 in the Drosophila circadian clock. Cell *105*, 769-779.

S103. Harms, E., Young, M.W., and Saez, L. (2003). CK1 and GSK3 in the Drosophila and mammalian circadian clock. Novartis Foundation symposium *253*, 267-277; discussion 102-269, 277-284.

S104. O'Neill, J.S., van Ooijen, G., Dixon, L.E., Troein, C., Corellou, F., Bouget, F.Y., Reddy, A.B., and Millar, A.J. (2011). Circadian rhythms persist without transcription in a eukaryote. Nature *469*, 554-558.

S105. van Ooijen, G., Hindle, M., Martin, S.F., Barrios-Llerena, M., Sanchez, F., Bouget, F.Y., O'Neill, J.S., Le Bihan, T., and Millar, A.J. (2013). Functional analysis of Casein Kinase 1 in a minimal circadian system. PLoS One *8*, e70021.

S106. Tan, S.T., Dai, C., Liu, H.T., and Xue, H.W. (2013). Arabidopsis casein kinase1 proteins CK1.3 and CK1.4 phosphorylate cryptochrome2 to regulate blue light signaling. The Plant cell *25*, 2618-2632.

S107. Gorl, M., Merrow, M., Huttner, B., Johnson, J., Roenneberg, T., and Brunner, M. (2001). A PEST-like element in FREQUENCY determines the length of the circadian period in Neurospora crassa. The EMBO journal *20*, 7074-7084.

S108. Huang, G., Chen, S., Li, S., Cha, J., Long, C., Li, L., He, Q., and Liu, Y. (2007). Protein kinase A and casein kinases mediate sequential phosphorylation events in the circadian negative feedback loop. Genes Dev *21*, 3283-3295.

S109. He, Q., Cha, J., He, Q., Lee, H.C., Yang, Y., and Liu, Y. (2006). CKI and CKII mediate the FREQUENCY-dependent phosphorylation of the WHITE COLLAR complex to close the Neurospora circadian negative feedback loop. Genes Dev *20*, 2552-2565.

S110. Tataroglu, O., Lauinger, L., Sancar, G., Jakob, K., Brunner, M., and Diernfellner, A.C. (2012). Glycogen synthase kinase is a regulator of the circadian clock of Neurospora crassa. J Biol Chem *287*, 36936-36943.

S111. van Dijken, J.P., Bauer, J., Brambilla, L., Duboc, P., Francois, J.M., Gancedo, C., Giuseppin, M.L., Heijnen, J.J., Hoare, M., Lange, H.C., et al. (2000). An interlaboratory comparison of physiological and genetic properties of four Saccharomyces cerevisiae strains. Enzyme and microbial technology *26*, 706-714.
